# Global transcriptional activity dynamics reveal functional enhancer RNAs

**DOI:** 10.1101/gr.233486.117

**Published:** 2018-12

**Authors:** Yoon Jung Kim, Peng Xie, Lian Cao, Michael Q. Zhang, Tae Hoon Kim

**Affiliations:** 1 Department of Biological Sciences and Center for Systems Biology, University of Texas at Dallas, Richardson, Texas 75080, USA

## Abstract

Active enhancers of the human genome generate long noncoding transcripts known as enhancer RNAs (eRNAs). How dynamic transcriptional changes of eRNAs are physically and functionally linked with target gene transcription remains unclear. To investigate the dynamic functional relationships among eRNAs and target promoters, we obtained a dense time series of GRO-seq and ChIP-seq data to generate a time-resolved enhancer activity map of a cell undergoing an innate antiviral immune response. Dynamic changes in eRNA and pre-mRNA transcription activities suggest distinct regulatory roles of enhancers. Using a criterion based on proximity and transcriptional inducibility, we identified 123 highly confident pairs of virus-inducible enhancers and their target genes. These enhancers interact with their target promoters transiently and concurrently at the peak of gene activation. Accordingly, their physical disassociation from the promoters is likely involved in post-induction repression. Functional assessments further establish that these eRNAs are necessary for full induction of the target genes and that a complement of inducible eRNAs functions together to achieve full activation. Lastly, we demonstrate the potential for eRNA-targeted transcriptional reprogramming through targeted reduction of eRNAs for a clinically relevant gene, *TNFSF10*, resulting in a selective control of interferon-induced apoptosis.

Enhancers are key *cis*-regulatory elements that play an essential role in genome expression to determine cell fates and functions. There are millions of enhancers in the human genome, and these enhancers function to shape cell identity by directing distinct genome expression programs. In practice, these enhancers can be systematically identified by the presence of histone modification of H3K4me1 (Heintzman et al. 2007, 2009) and H3K27ac (Rada-Iglesias et al. 2011), the association of transcription factors and coactivators (Heinz et al. 2015), and/or DNase I hypersensitivity (The ENCODE Project Consortium 2007; Thurman et al. 2012). Functional hierarchies among these enhancers have been described (Ernst and Kellis 2010). Recently, enhancers were found to be transcriptionally active and generate noncoding RNAs known as enhancer RNAs (eRNAs) as relatively unstable transcripts (Kim et al. 2010; Wang et al. 2011). Several studies have demonstrated eRNA-producing enhancers are more potent and associated with higher expression of nearby genes than enhancers without eRNAs (Wang et al. 2011; Heinz et al. 2015; Romanoski et al. 2015) and transcriptional activity at enhancers precedes target gene expression (Arner et al. 2015). Thus, eRNA-producing enhancers are likely active and functional enhancers that define the identity and function of a given cell. Moreover, targeting enhancer activity for therapeutic development has been recently proposed and pursued by several groups and companies (Bradner et al. 2017). By targeting particular enhancers, disease-specific modulation of gene expression would be possible without affecting the normal expression in other tissues and organs. However, for the over two million enhancers that have been annotated (Roadmap Epigenomics Consortium et al. 2015), currently only tens of thousands of eRNAs have been detected in the human genome through isolated studies (Li et al. 2016). A systematic detection and annotation of eRNAs is necessary to enable functional characterization of eRNA gene regulation, which is a fundamental step toward therapeutic development.

In-depth studies of eRNAs in regulation of key biological processes require accurate prediction of target genes. Existing methods are mostly based on eRNA and mRNA levels in steady-state cells, which may not provide enough information for functional associations. Active enhancers may have multiple nearby genes and vice versa, but functionally associated pairs will be triggered to be transcriptionally active in a synchronized fashion. Thus, eRNA/pre-mRNA dynamics, induced by a stimulus, may represent a highly informative feature for more reliable enhancer target predictions (Arner et al. 2015). For example, in our previous study (Banerjee et al. 2014), we took advantage of the dynamic physical chromatin interactions to identify a functional enhancer responsible for the *IFNB1* gene, a critical component of innate and adaptive immunity.

In order to systematically investigate the functionality of eRNAs in the human genome, we have employed a battery of comprehensive, unbiased functional genomic experiments across multiple time points to annotate and investigate the dynamics of enhancer and target gene activation. We also design a novel computational strategy for determining functional eRNAs that are virus-inducible and mediate innate anti-viral response. Combined with functional assessment using RNAi and time course chromosome conformation capture (3C), we examine functional relevance of these virus-inducible eRNAs, their regulatory trajectories, and modes of action.

## Results

### A time-resolved enhancer activity map

**Figure 1.**
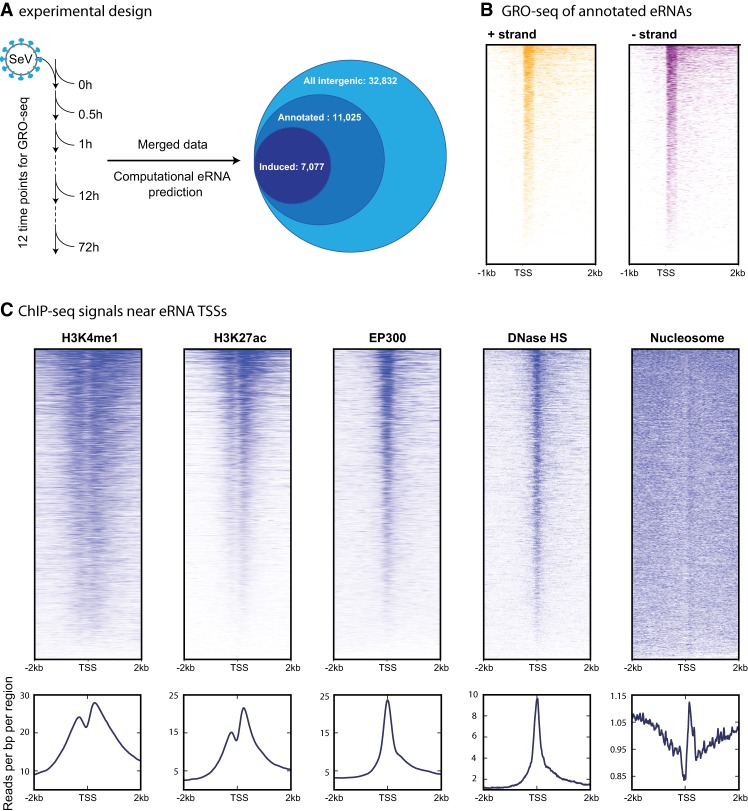
Genome-wide eRNA identification. (*A*) Venn diagram shows the number of all the intergenic transcribed regions (*outer* circle, light blue), high confidence enhancer regions (*middle* circle, blue), and inducible enhancer regions (*inner* circle, dark blue). (*B*) Heat map summarizes GRO-seq data in eRNA-TSS flanking regions (from 1 kb upstream to 2 kb downstream). eRNAs from + (yellow) and − (purple) strands are shown separately. Predicted eRNA-expressing enhancer regions are centered at the TSS. (*C*) Heat map (*upper* panel) and metagene profiles (*lower* panel) are plotted for epigenetic signals including H3K4me1, H3K27ac, and EP300 ChIP-seq, DNase-seq, and MNase-seq data.

In order to obtain the informative features of eRNA/mRNA dynamics, we performed a large time series GRO-seq analysis of B-lymphoblasts (GM12878) during innate anti-viral immune response. We used Sendai Virus (SeV) to activate the immune response signal-cascade gene induction system as a model to study the anti-viral program. We first combined all GRO-seq data obtained from 12 time points from 0 to 72 h post-infection to determine a compendium of eRNA-producing enhancers responding to virus, then used HOMER (Heinz et al. 2010) to identify the eRNA transcripts (see Methods). Of 32,832 total intergenic transcripts, 11,025 transcripts overlapped with H3K4me1 or H3K27ac histone modification peaks, representative enhancer marks (Fig. 1A). We annotated transcription start/termination sites (TSSs/TTSs) for the 11,025 eRNAs (Fig. 1B). The average predicted length of eRNAs from our annotation efforts was 1746 bp (Supplemental Fig. S1A). Other enhancer marks including EP300 and DNase hypersensitivity signals were highly enriched at the TSSs of eRNAs (Fig. 1C; Lai and Pugh 2017). Additionally, the patterns of H3K4me1 and H3K27ac ChIP-seq, DNase hypersensitivity and MNase-seq indicated an open chromatin region at the eRNA TSSs. Notably, a majority (64%) of the eRNAs were below detection levels prior to virus infection, thereby indicating a dramatic induction of eRNA synthesis upon virus infection (Fig. 1A). We have compared the resulting eRNA annotation to the previous large-scale analysis of 43,012 eRNAs obtained from 69 human cell types by the FANTOM consortium (Andersson et al. 2014). Only ∼28% of our eRNA annotations overlapped with that of the FANTOM5 human enhancer atlas (Andersson et al. 2014), underscoring condition-specific differences. In addition, we found 18,999 intergenic transcripts, lacking initial eRNA production and enhancer marks, being transcribed after infection. These transcripts showed similar lengths as eRNAs defined above (*P*-value 0.14, *t*-test). A previous study showed that enhancers initially lacking known enhancer marks, like the 18,999 enhancers that we found here, could acquire enhancer-associated epigenetic modifications upon stimuli (Kaikkonen et al. 2013).

### Rapid and dynamic transcriptional response of genes and enhancers

**Figure 2.**
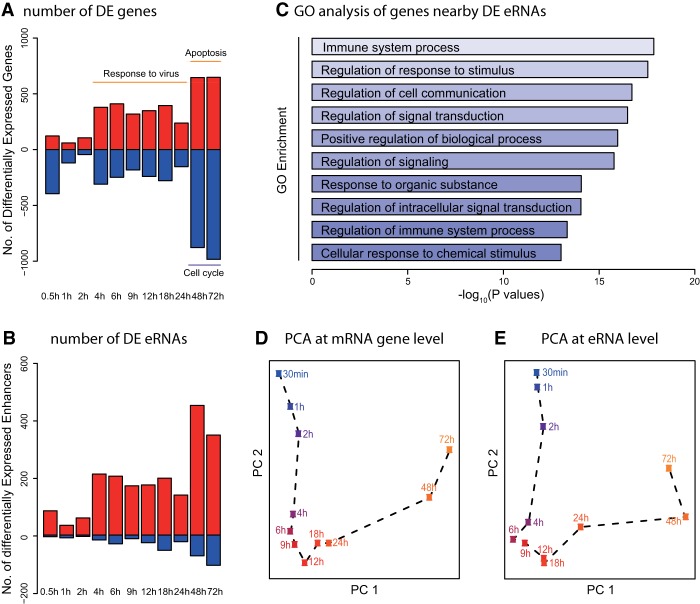
Analysis of differentially expressed genes. (*A*) Number of up- (red) and down- (blue) regulated genes in time course after virus infection. (*B*) Number of up- (red) and down- (blue) regulated enhancers through time course. (*C*) Bar plot of GO terms enriched in the nearest genes linked to the inducible enhancers. (*D*,*E*) First two principal components of PCA analysis based on mRNA (*D*) and eRNA (*E*) expression level. The plots are color-coded by time points: blue (early) and orange (late).

We quantified expression levels of the RefSeq genes and performed differential expression (DE) analysis. Based on the expression dynamics of DE genes (Fig. 2A), the time course can be divided into three stages: 0–2 h, limited changes; 4–24 h, significant changes with more induced genes (early-up) than repressed; 48–72 h, large changes comprised of both up- (late-up) and down- (late-down) regulated genes. To understand these expression dynamics more meaningfully, we performed Gene Ontology (GO) analysis at each time point (Supplemental Fig. S1B). DE gene-enriched functions were highly consistent between time points within each of the three stages. For example, the most frequently enriched GO terms of early-up, late-up, and late-down groups were “responses to virus,” “apoptosis,” and “cell cycle,” respectively. We also performed DE analysis with the annotated eRNAs. Their expression dynamics could also be divided into three stages, exactly matching those of DE genes (Fig. 2B). Representative examples of inducible genes and eRNAs are shown in Supplemental Figure S1C,D. Furthermore, nearest genes of the inducible eRNAs (described in the section describing enhancer-promoter pairs) were functionally enriched in immune system processes (Fig. 2C).

To visualize and verify the dynamics of eRNA and gene expression patterns, we performed principal component analysis (PCA), which showed a trajectory of cellular states (Fig. 2D,E). The first two principal components (PCs) clearly separated samples from each time points. Principal component 2 (PC2) values showed an interesting trajectory, which moved away from the baseline in early time and returned after 18 h, matching the expression dynamics of immune related genes (GO term “defense response to virus,” *P*-value = 2.5 × 10^−12^). Similar analysis was performed for genes correlated with PC1, showing enrichment of GO terms “translation,” “apoptosis,” and “RNA decay.” Likewise, the first two PCs of eRNAs showed similar dynamics as those of gene expression, indicating connected regulatory processes between eRNAs and gene expression. In addition, t-distributed stochastic neighbor embedding (t-SNE) (Jamieson et al. 2010) results showed almost identical patterns (Supplemental Fig. S1E,F).

### Cytokine *IFNB1* as a representative transient transcript

As a representative virus-inducible case, we investigated the *IFNB1* gene and its enhancer *L2*, which we previously identified as a novel virus-inducible long-range enhancer regulating *IFNB1* transcription in IMR-90 lung fibroblasts (Banerjee et al. 2014). An independent study (Decque et al. 2016) has also demonstrated that the *L2* is a major enhancer regulating *IFNB1* expression in bone marrow–derived dendritic cells and macrophages. Our GRO-seq data in GM12878 cells indicate strong transcription at *IFNB1* and the *L2* element in early time points just after SeV infection (Supplemental Fig. S2). *L2* eRNA was transcribed first at 1 h, and then *IFNB1* transcript emerged around 1 h after *L2*, implying that eRNA generation precedes target gene transcription. *L2* transcription continues to be detected even at 72 h post-infection when *IFNB1* has become repressed by post-induction repression mechanisms (Ren et al. 1999), implicating a potentially novel enhancer inactivation and decommissioning mechanism. We also investigated other well-known transcription factors of IRF and NFκB families which are also up-regulated at these earliest time points (Supplemental Fig. S2B).

### Construction of an enhancer-target gene map for viral response

**Figure 3.**
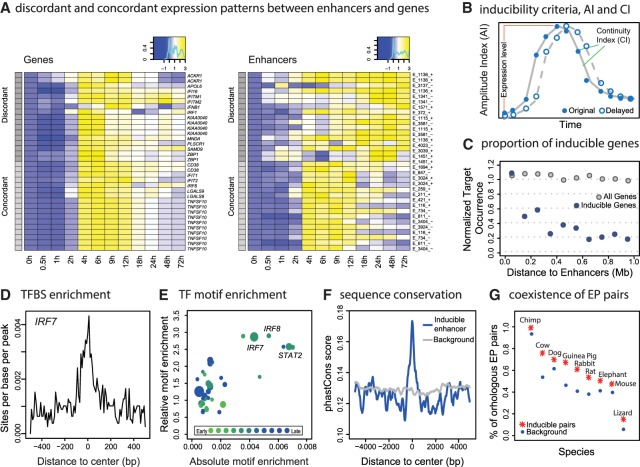
Prediction of virus-inducible enhancer-promoter (EP) pairs and validation of their interactions. (*A*) Heat map of discordant and concordant expression pairs of target genes (*left* panel) and their enhancers (*right* panel). Rows are matched. Expression levels were normalized as log_2_ fold changes relative to 0 h. Gray scales represent discordant (dark gray) and concordant (light gray) groups. (*B*) Diagram describes the identification of inducible enhancers and genes with two indices: the continuity index (CI) and the amplitude index (AI). (*C*) Number of inducible genes as a function of EP distance is analyzed. Inducible genes were counted within each 100-kb bin of inducible enhancers (blue points). As a control, the number of all genes in each bin was calculated (gray points). Each group was normalized by the maximum count for the sake of comparison. (*D*) Enrichment of TF *IRF7* motif in the 1-kb TSS-flanking region of inducible enhancers is shown. (*E*) Motif enrichment of inducible enhancers. *x*-axis (absolute enrichment) is the maximum sites per base per peak (SBP). *y*-axis (relative enrichment) is the SBP ratio between the center (−100 to 100 bp) and rest (−500 to −100 bp and 100 to 500 bp) of the flanking regions. Point sizes indicate the GRO-seq RPKM fold changes of TFs. Colors indicate the first time point when the TF reaches half induction. (*F*) Average phastCons conservation scores of primates across inducible enhancer regions are shown, relative to randomly selected background. (*G*) Percentage of inducible human EP pairs that co-exist in other species is shown.

Accurate cell-specific and genome-wide enhancer target identification is a challenging task. Despite several improvements in the past few years (Jin et al. 2013; Whalen et al. 2016; Cao et al. 2017), the accuracy is still far from satisfactory for in-depth case studies of individual genes or enhancers. Using metagene analysis, our results, as well as several other studies (Hah et al. 2013; Kaikkonen et al. 2013), have shown the coordinated transcriptional dynamics of enhancers and neighbor genes should be highly enriched with functional targets. Can we take advantage of the paired expression profiles to further refine target prediction for individual enhancers? To this end, we carefully examined the expression profiles of eRNA/gene pairs that were significantly activated by SeV infection. In addition to the expected, correlated pattern of concordant on/off behaviors between enhancers and genes, we also observed a discordant expression pattern showing persistent eRNA transcription after target gene repression (Fig. 3A). This discordant pattern was exhibited by the previously validated *L2*-*IFNB1* EP (enhancer-promoter) pair. Thus, the co-inducibility of eRNAs and target genes is a potentially important feature for inferring functional enhancer targets, regardless of regulatory divergence of the concordant and discordant sets of eRNA/gene pairs. To identify inducible enhancers and genes, we constructed two indices: the continuity index (CI) and the amplitude index (AI) (Fig. 3B; see Methods). The CI is used to filter out random fluctuations in the expression levels, especially for eRNAs which are lowly expressed and more subject to technical variability. The AI is designed to represent the maximum induced levels, which is stable with respect to the specific expression patterns but can be highly variable for each EP pair. In these indices, 299 genes and 787 enhancers were identified as inducible. Consistent with the induced transcriptional activity, the H3K27ac levels of these 787 enhancers were also induced by SeV infection (Supplemental Fig. S1G).

Genomic proximity is also an important factor for identifying enhancer targets (Sanyal et al. 2012). We found that inducible genes were highly enriched within 200 kb of the inducible enhancers (Fig. 3C) and vice versa (Supplemental Fig. S3A,E). We assigned the inducible enhancers with the nearest inducible genes within 200 kb and obtained 123 highly confident enhancer-promoter (EP) pairs (Supplemental Table S1). Extending the proximity window farther enabled us to define more enhancer-promoter pairs, but this increased sensitivity of identifying more EP pairs also resulted in significant increases in the false positive rate for our inducible EP prediction. For example, the percentage of inducible genes decreased from ∼50% to ∼30% if the distance threshold was increased by another 100 kb. In addition, our current analysis focused only on the mRNA encoding target genes and excluded possible target genes encoding noncoding RNAs due to the limited functional information regarding these genes. This highly prioritized inducible eRNA and target gene set included not only the previously validated *L2*-*IFNB1* pair but also other critical genes involved in immune function, such as the *CD38*, *IRF8*, *TNFSF10*, and *TLR7* genes, whose distal regulatory elements were largely unknown.

### Inducible enhancers have conserved sequences

Since enhancers activate their target genes by recruiting transcriptional factors (TFs), we hypothesized that these inducible enhancers might have distinct TF binding motifs supporting virus-inducible gene regulation. We analyzed human TF motif occurrences within the inducible enhancers and found a strong enrichment of binding sites for IRF- and STAT-family proteins, which are known interferon-responsive factors (Fig. 3D,E). In addition, *IRF7* and *STAT2* were up-regulated by more than twofold post-SeV treatment (Fig. 3E). Most TFs with high motif enrichment reached 50% of their maximum expression levels no later than 6 h after virus infection (Fig. 3E), thus supporting our hypothesis of enhancer induction through TF activation.

These inducible enhancers showed a significantly higher evolutionary sequence conservation level than carefully selected background regions (Fig. 3F; Supplemental Fig. S3B,C), especially near the TSSs of eRNAs. Synteny of enhancer and promoters across 11 species spanning the vertebrate phylogenetic tree was also examined (see Methods). We found that the induced EP pairs had ∼10% higher chance than the random background to be immobilized on the same chromosome across the 11 vertebrate genomes (Fig. 3G). Moreover, the induced pairs showed a higher probability to remain in close proximity to each other (<500 kb) (Supplemental Fig. S3D).

### Dynamic physical EP association correlated with target gene expression patterns

**Figure 4.**
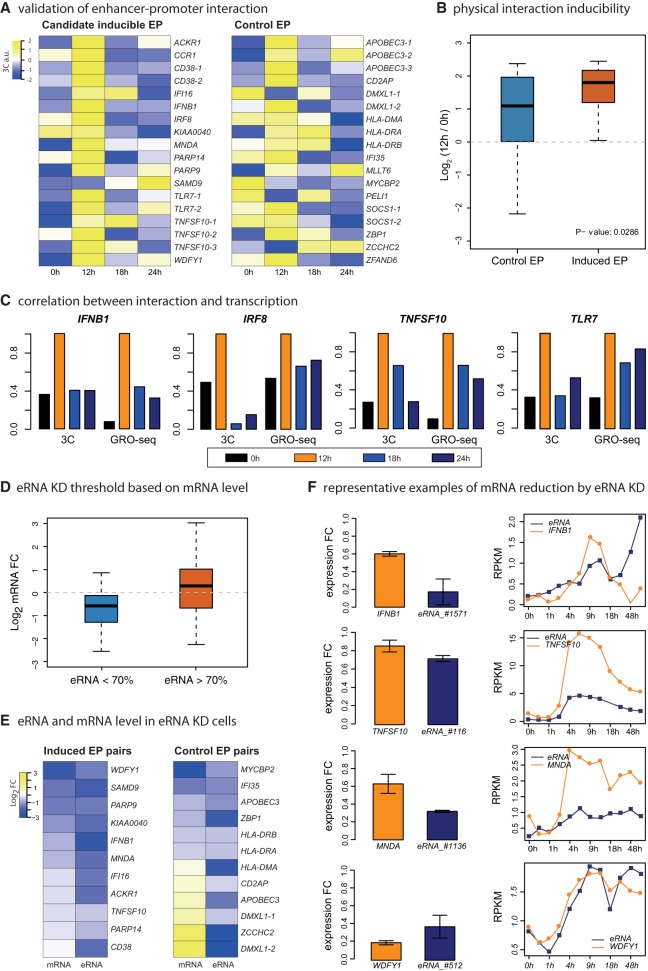
Effects of eRNA KD on target genes. (*A*) Heat maps of 3C signals for 18 inducible EP pairs and 18 control EP pairs within 200 kb are shown. Signals are normalized by BAC 3C interaction frequency. (*B*) Box plot of 3C log fold changes is shown (12 h vs. 0 h) for control and induced EP pairs with *P*-values from Kolmogorov–Smirnov (KS) test. (*C*) Representative examples of 3C results are shown and compared with GRO-seq signal (RPKM) of the corresponding immune-related genes. (*D*) Box plot shows mRNA fold changes for successful eRNA KD (eRNA < 70%) and unsuccessful eRNA KD (eRNA > 70%). (*E*) Heat map shows mRNA/eRNA log fold changes; 11 inducible EP pairs and 12 control pairs are included. (*F*) Representative examples of EP pairs where eRNA KD led to mRNA repression. mRNA/eRNA expression levels were measured by RT-qPCR. *y*-axes of expression curves represents GRO-seq signal (RPKM).

Physical interaction between an enhancer and its target promoter has been accepted as a general mechanism of gene activation. It is thought that many inducible genes are regulated through pre-existing interactions with enhancers (Jin et al. 2013); however, the fate of these interactions after induction when the gene is turned off has not been adequately addressed. We examined the dynamic physical interaction of 18 inducible EP pairs using a time course chromosome conformation capture assay. We also sampled 18 active enhancers and genes within 200 kb that did not pass the inducibility criterion as a control set (Supplemental Tables S2, S3). Most inducible EP pairs showed the highest interaction between enhancer and promoter at 12 h (Fig. 4A). This transient physical interaction correlated with the corresponding target gene expression profiles (Fig. 4C). In contrast, the control pairs showed highly variable interaction patterns during the time course and a lower inducibility of physical interaction (Fig. 4A,B). The inducible EP pairs showed a significantly higher inducible interaction frequency (Kolmogorov–Smirnov test, *P*-value = 0.0286) (Fig. 4B). A notable observation from this analysis is that, in most cases, a decrease in physical interaction after 12 h post-infection coincided with a concomitant decrease in target gene transcription. Thus, this transient physical EP interaction may determine the maximal promoter activity. In addition, some eRNAs can continue to be transcribed beyond 12 h post-infection, as in the case for the *IFNB1* and *L2* and many other EP pairs. Therefore, physical dissociation of an enhancer from its target promoter might be a critical mechanism of post-induction repression of these target genes, irrespective of the status of eRNA synthesis at the enhancers.

### Functional relevance of eRNA expression in target gene activation

Although many eRNAs have been shown to be important for target promoter regulation by a number of studies (Li et al. 2013; Melo et al. 2013; Mousavi et al. 2013), there is an emerging debate concerning the general functionality of eRNAs from several studies that suggest eRNAs are dispensable for enhancer function (Hah et al. 2013; Kaikkonen et al. 2013; Engreitz et al. 2016; Rahman et al. 2017). To examine the functional relevance of the inducible eRNAs that we have identified, we employed systematic siRNA-mediated eRNA knockdown (KD) assays and determined their target gene expression before and after siRNA transfection. In total, we used 85 siRNAs for depleting eRNAs of both induced and control EP pairs (Supplemental Tables S5–S7). Forty-nine siRNAs were able to reduce eRNA expression levels from 28 enhancers (eRNA fold change [eFold] < 1) (Supplemental Fig. S4; Supplemental Table S7). We performed statistical analysis and determined eFold < 0.7 as a reasonable threshold for assessing the effectiveness of eRNA KD experiments (Fig. 4D). Eleven inducible and 12 control EP pairs that passed this threshold were further examined. All target genes in the inducible EP pairs were repressed by eRNA KD (Fig. 4E, left). In contrast, half of the target genes from the control EP pairs were not repressed, and some were even activated upon eRNA reduction (Fig. 4E, right). Representative cases from the inducible EP pairs are shown in Figure 4F. Similar to our 3C results, this knockdown analysis of eRNAs demonstrates that the inducible eRNAs are biochemically functional in mediating target gene activation.

### Inducible eRNAs promote physical interaction with target promoters

**Figure 5.**
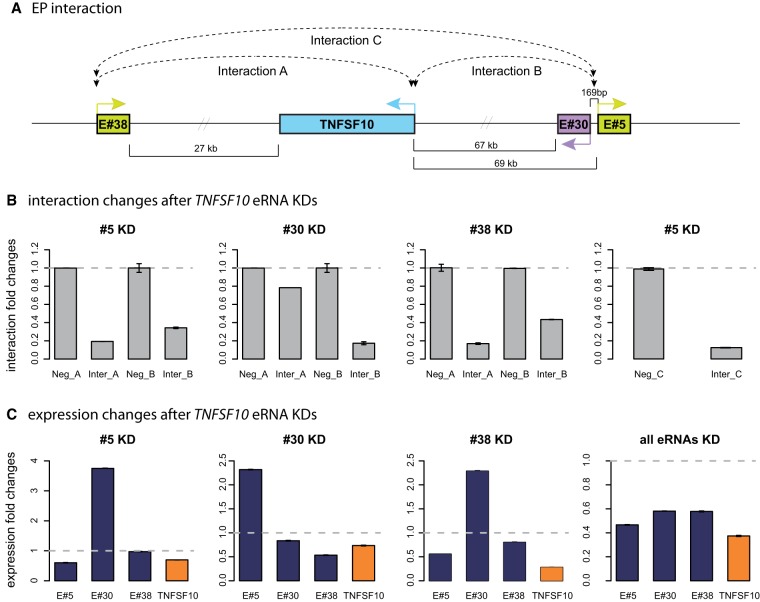
Effects of multiple enhancers on target gene expression and chromosomal conformation. (*A*) Schematic diagram of *TNFSF10* gene with its multiple enhancers. *TNFSF10* has #38 enhancer 27 kb upstream, and #30 and #5 enhancers 67 and 69 kb downstream, respectively. Colored arrows from the gene and enhancer indicate transcriptional direction. Dashed lines show physical interaction between promoter and enhancer (Interaction A and B) or between different enhancers (Interaction C). (*B*) Interaction changes between enhancer and promoter regions (Interaction A and B) after individual eRNA KD and all three combined eRNA KD. (*C*) Three eRNAs (dark blue) and target gene (orange) expression fold changes after each individual eRNA KD and after all three eRNA KD by combining three corresponding siRNAs.

According to our current 3C results and the results of previous reported studies (Banerjee et al. 2014; Schaukowitch et al. 2014), the transient physical association pattern was highly correlated with the transient transcription pattern of the target genes (Fig. 4A). Also, the eRNA KD results indicated functional relevance of these inducible eRNAs in the target gene transcription (Fig. 4E). Based on these results, we investigated if eRNAs play a general role in mediating physical interactions. We first analyzed the physical higher order chromatin interactions by 3C assay upon eRNA KD of the *IFNB1* gene. This led to decreased physical interaction of the enhancer with the promoter by about 20% (Supplemental Fig. S5A). We also examined the *TNFSF10* locus containing three distinct inducible enhancers, enabling us to examine the effect of single eRNA KD on multiple EP interactions. Single eRNA KD led to dissociation of the corresponding enhancer from the promoter, as well as reduced interaction between the other enhancers and the *TNFSF10* promoter (Interaction A-B in Fig. 5A,B) and among the three enhancers (Interaction C in Fig. 5A,B). The *IFI35* and *MYCBP2* EP pairs were analyzed as control pairs (Supplemental Fig. S5B,C). Their eRNA KD did not affect the interaction between the enhancer and the promoter. *MYCBP2* eRNA KD resulted in an increase of EP interaction and the elevated target gene expression levels. This observation was not a unique case, as we have identified a number of eRNAs with similar functional profiles (Fig. 4E), suggesting there may be diverse classes of eRNAs (i.e., activator- and repressor-eRNAs). Results from these targeted studies suggest that inducible eRNAs exhibit a strong physical and functional association with the target genes. In contrast, noninducible eRNAs exhibit much weaker functional and physical association with the target genes.

### Multiple eRNAs collaborate in regulating target gene transcription

Since genes can be regulated by a combination of multiple enhancers (Joo et al. 2016), we asked how might multiple inducible eRNAs coordinate their action on their target gene. We performed combinatorial eRNA KD by applying combined siRNAs to determine how eRNAs may function together. We examined the *TNFSF10* gene, which has three inducible enhancers based on our analysis (#5, #30, and #38, Fig. 5A). Single eRNA KD decreased EP interaction and *TNFSF10* transcription (Fig. 5B,C). However, the effects on the levels of other eRNAs showed a complex pattern (Fig. 5C): #5KD reduced #38 but increased #30; #30KD decreased #38 but increased #5; #38KD reduced #5 but increased #30. One clear pattern from this analysis is that there is a reciprocal and compensatory relationship between #5 and #30 eRNAs, which are bidirectional divergent transcripts originating from a single enhancer. This reciprocal effect was also observed in our previous work on the *L2* enhancer (Banerjee et al. 2014). When all three siRNAs were combined, all three eRNAs decreased, as well as the target *TNFSF10* mRNA (Fig. 5C). We also analyzed the *TLR7* and *CD38* genes, each with two inducible eRNAs (Supplemental Fig. S6). Overlapping bidirectional eRNAs from *TLR7* also showed the reciprocal effect under single eRNA KD. In the case of *CD38*, a distal eRNA (#37) seems to be more dominant than a more proximal eRNA (#17) in its contribution to the target gene activation. For both *TLR7* and *CD38*, double KD of eRNAs reduced the corresponding mRNA expression incrementally. Taken together, these targeted analyses demonstrate how inducible eRNAs collaborate to support their target gene transcription. Overlapping, bidirectional eRNAs represent an interesting class of eRNAs displaying a compensating expression pattern upon knockdown and likely serve redundant roles to maintain the target gene expression. In addition, the transcriptional direction of eRNAs does not seem to be an important factor in determining their functional contribution to target gene expression.

### Targeting *TNFSF10* eRNA activity limits apoptosis

**Figure 6.**
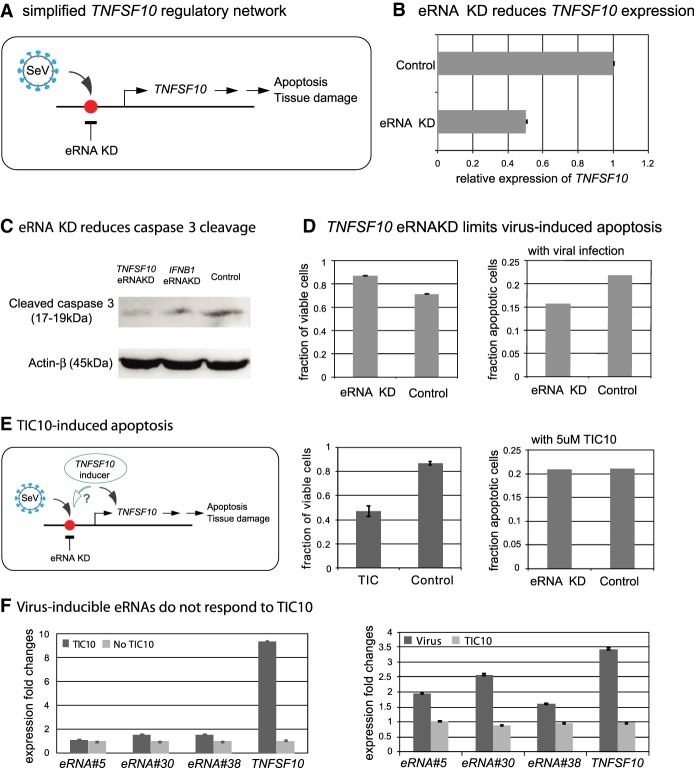
Effect of selective inhibition of *TNFSF10* by eRNA knockdown on virus-induced apoptosis. (*A*) Simplified representation of the *TNFSF10* regulatory network. (*B*) Relative expression of *TNFSF10* in eRNA knockdowns (#5, #30, and #38, triple eRNA KD) and control cells. Triple KD of eRNAs regulating the *TNFSF10* results in loss of *TNFSF10* expression. (*C*) Western blot using cleaved caspase 3 antibody to assess apoptosis. Stronger signal of cleaved caspase 3 indicates higher fraction of apoptotic cells. As an internal standard, beta actin was used. For an uncropped image of these blots, please see Supplemental Figure S8. (*D*) Cell viability by live cell counts (*left* panel) and apoptotic cell counts (*right* panel) for *TNFSF10* eRNA KD and control cells are shown. (*E*) Regulation of apoptosis by TIC10- or virus-induced *TNFSF10* expression. Possible mechanism through eRNA or direct gene activation is shown in the *left* panel. Cell viability by live cell counts (*middle* panel) for TIC10-treated cell, control cell, and apoptotic cell counts (*right* panel) for eRNA KD (triple KD) and control KD upon TIC10 treatment for 48 h. (*F*) *TNFSF10* and its three eRNAs expression fold changes with or without TIC 10 treatment for 48 h (*left* panel), and with SeV or TIC10 treatment for 9 h (*right* panel).

Thus far, our study has identified a validated set of functional eRNAs and established that modulation of these eRNAs can yield selective changes in target gene expression. These findings could be valuable for a therapeutic intervention by targeted enhancement or reduction of disease-relevant genes. In the context of the anti-viral response in human and mouse, overexpression of the *TNFSF10* gene has been implicated in inducing lung damage by influenza virus (Hogner et al. 2013). We reasoned that, by targeted reduction of *TNFSF10* eRNAs to decrease *TNFSF10* expression, we may be able to limit apoptosis without affecting interferon production or response. In order to examine the possibility of eRNA modulation for reducing apoptosis, we performed siRNA-mediated reduction of *TNFSF10* eRNAs and checked cell viability (Fig. 6A). *TNFSF10* expression level, upon triple eRNA KD, decreased about 50%, compared to the control (Fig. 6B). Control cells showed a stronger signal for cleaved caspase 3 than the *TNFSF10* eRNA KD cells and a positive control, *IFNB1* eRNA KD cells (Fig. 6C; Supplemental Fig. S8). Furthermore, reduction of *TNFSF10* eRNA resulted in a higher proportion of live cells and a corresponding decrease in apoptotic cells (Fig. 6D). To confirm that *TNFSF10* eRNA KD is specific for limiting the virus-induced apoptosis, we induced *TNFSF10* expression by a distinct mechanism using TIC10, a small molecule inducer of *FOXO3* that activates the *TNFSF10* promoter (Jacob et al. 2014). Thus, TIC10 treatment would result in apoptosis via a distinct mechanism, compared to viral infection-induced apoptosis. As expected, *TNFSF10* eRNAs KD, either individual KD or triple KD, did not affect TIC10-induced apoptosis (Fig. 6E; Supplemental Fig. S7). In addition, TIC10 treatment induced *TNFSF10* expression significantly, but it did not affect expression of the virus-inducible eRNAs associated with the *TNFSF10* gene (Fig. 6F, left). In contrast, virus infection induced expression of those eRNAs as well as the *TNFSF10* gene (Fig. 6F, right). Taken together, these results demonstrate that targeted reduction of eRNAs can specifically inhibit interferon-induced apoptosis.

## Discussion

Regulatory genomic elements outnumber genes by two orders of magnitude. More than two million enhancers have been annotated in the human genome (Romanoski et al. 2015). One fundamental question is whether all these enhancers are functionally equivalent or whether there are distinct classes of enhancers that are more relevant in different conditions. To explore the landscape of potentially functional enhancers and associated eRNAs, we used the virus-inducible gene expression model. We identified potentially functional eRNAs by taking advantage of the dynamic expression information from global transcriptional analysis and using associated genomic proximity and transcriptional activity as criteria. Specifically, we considered inducibility of enhancers upon virus infection and applied this activity-based association strategy to identify their target gene. From this strategy, we were able to assign 123 eRNAs to their most likely target genes. Using these highly confident EP pairs, we tested the functionality of eRNAs at both the physical interaction level and biochemical level. More than 80% of the inducible EP pairs showed a higher physical interaction frequency at 12 h—a time point representing the peak of transcription level. In addition, reduction of eRNA levels by RNAi decreased target gene transcription for all inducible eRNAs tested. From these results, we conclude that the virus-inducible eRNAs are indeed functional. The control pairs for our experiments were selected by general criteria that other groups have routinely used for determining active enhancers. Our 3C results and targeted eRNA reduction results from control sets might explain why many groups have argued that eRNAs are dispensable for enhancer function. Thus, co-inducibility of eRNAs and genes would be relevant for identifying other functional eRNAs in different biological conditions.

One unexpected finding from our study is that the fate of an enhancer can be different from the promoter it regulates after their transient functional and physical association. In many independent cases, eRNA production continues while the enhancer becomes disengaged from its associated promoter and the target gene undergoes post-induction repression. In other cases that conform to the current paradigm of gene regulation, when an enhancer disengages from its promoter, both eRNA and mRNA production are shut off concordantly. Furthermore, dependence of eRNAs for physical interaction between enhancers and promoters does not seem to be a universal mechanism across different loci (Li et al. 2013; Schaukowitch et al. 2014). Rather, each locus exhibits different dependencies on eRNAs for enhancer-promoter interaction. Notably, some loci display competition among enhancers and promoters when assayed for physical interaction. In reduced-function assays using RNAi, enhancers within the same locus can also compete for production of corresponding eRNAs. Despite the complex regulatory dependencies among enhancers and promoters, a combined reduction of all eRNAs for a given target gene resulted in the largest decrease in target gene expression compared to individual eRNA knockdowns, suggesting a complete pool of functionally relevant eRNAs is necessary for proper regulation. Thus, the human genome displays dynamic and complex exchanges of physical and functional associations among enhancers and promoters to define genome expression. These functional properties of eRNAs are consistent with a recently proposed model of RNA-mediated phase separation for gene regulation (Hnisz et al. 2017).

Lastly, we demonstrated that we can modulate one particular enhancer of the anti-viral program to achieve a specifically modified cellular behavior that can aid in reducing excessive inflammation. With hundreds of functional eRNAs identified in this study, targeted therapies with tailored modulation of multiple enhancers may be an approach to achieve a personalized clinical response.

## Methods

### Cell culture and virus infection

B-Lymphoblasts, GM12878, were obtained from Coriell Institute for Medical Research and cultivated according to the supplier's instructions. Fifteen percent fetal bovine serum was added to Roswell Park Memorial Institute media 1640 (RPMI-1640) with 2 mM L-glutamine for the culture. Sendai Virus (Cantrell strain) obtained from Charles River was used for inducing anti-viral immune response—50 µL of viral stock was added to 1 mL media. Cell samples were taken at 30 min, 1, 2, 4, 6, 12, 18, 24, 48, and 72 h after virus infection for the GRO-seq experiment. For the other experiments, 3C assay and the ChIP-seq experiment, the cells incubated for 6, 12, 18, and 24 h after infection were sampled. Untreated GM12878 cells were used as a control, which is the 0h sample.

### GRO-seq analysis

Global run-on and library preparation for sequencing was performed based on the method published by John Lis et al. in 2008 (Core et al. 2008). To generate multi-indexing sequencing libraries, an Illumina TruSeq Small RNA Library Prep kit-set A (24 rxns, RS-200-0012) was used (Kim et al. 2013).

#### Nuclei isolation

Two 15-cm plates of confluent cells (∼10–20 million cells) were washed three times with ice-cold PBS buffer and incubated for 5 min with 10 mL cold swelling buffer (10 mM Tris-Cl at pH 7.5, 2 mM MgCl_2_, 3 mM CaCl_2_) for each plate, on ice. Cells were scraped from the plate, harvested, centrifuged at 500*g* for 10 min at 4°C and resuspended in 1 mL of lysis buffer (swelling buffer with 0.5% IGEPAL, 10% glycerol, and 4 U/mL SUPERaseIn) with gentle mixing by pipetting with a wide bore pipette tip up and down 20 times. For the isolation of nuclei, 9 mL of the same lysis buffer (up to total 10 mL) was added. After collection by centrifugation (at 300*g* for 5 min at 4°C), the nuclei were resuspended in 1 mL freezing buffer per 5 million nuclei, pelleted, and resuspended to a final volume of 100 µL (about 5–10 million nuclei/100 µL) of freezing buffer (50 mM Tris-Cl at pH 8.3, 5 mM MgCl_2_, 0.1 mM EDTA, 40% glycerol).

#### Nuclear Run*-*On (NRO)

Before the NRO reaction, NRO reaction buffer (10 mM Tris-Cl at pH 8.0, 5 mM MgCl_2_, 1 mM DTT, 300 mM KCl, 50 µM ATP, GTP and Br-UTP, 2 µM CTP, 0.4 U/µL RNasin, and 2% sarkosyl) was prepared and preheated to 30°C for 5 min. An equal volume (100 µL) of NRO reaction buffer was mixed with 100 µL of thawed nuclei solution in freezing buffer and was incubated at 30°C for 5 min with mixing at 800 rpm on a thermomixer. Then, RQ1 DNaseI (Promega) was added along with DNaseI reaction buffer and samples were incubated at 37°C for 20 min with mixing at 800 rpm. To stop the NRO reaction, 225 µL NRO stop solution was added to the reaction and 25 µL of Proteinase K was added. The sample was incubated for 1 h at 55°C. Nuclear RNA was extracted with acidic phenol (Sigma) and then with chloroform (Sigma) and was precipitated and washed. RNA was then resuspended in 20 µL of nuclease-free water and subjected to base hydrolysis by addition of 5 µL of 1 N NaOH on ice for 10 min. The reaction was neutralized with 50 µL of 0.5 M Tris-Cl at pH 6.8. Then, RNA was purified through a Bio-Rad P-30 RNase-free spin column following the manufacturer's instructions and was treated with 7 µL of DNaseI buffer and 3 µL RQ1 DNaseI (Promega) for 10 min at 37°C and purified again with a Bio-Rad P-30 column.

#### Br*-*UTP binding

Anti-BrdU (clone IIB5) agarose beads (Santa Cruz Biotech, sc-32323 AC) were equilibrated by washing them two times in 500 µL BrU binding buffer (0.25× SSPE, 1 mM EDTA, 0.05% Tween-20, 37.5 mM NaCl) and blocked in 1 mL BrU blocking buffer (1× binding buffer, 0.1% PVP, and 1 mg/mL BSA) for 1 h with rotation at 4°C. During the blocking step, beads were washed two times with 500 µL binding buffer, the NRO RNA sample was heated at 65°C for 5 min and then placed on ice for at least 2 min. Fifty microliters of the blocked bead mixture were combined with RNA sample in 450 µL binding buffer and mixed for 1 h by rotating at 4°C. After binding, beads were washed once in low-salt buffer (0.2× SSPE, 1 mM EDTA, 0.05% Tween-20), once in high-salt buffer (0.25× SSPE, 1 mM EDTA, 0.05% Tween-20, 137.5 mM NaCl), and twice in TET buffer (TE with 0.05% Tween-20). BrU-incorporated RNA was eluted four times with 100 µL elution buffer (20 mM DTT, 300 mM NaCl, 5 mM Tris-Cl at pH 7.5, 1 mM EDTA, and 0.1% SDS). RNA was then extracted and precipitated as described above. The precipitated RNA was resuspended in 20 µL of water.

#### TAP/PNK treatment

RNA was heated to 65°C for 5 min and cooled on ice for at least 2 min. The RNA was treated with TAP (by adding 3 µL 10× TAP buffer, 5 µL water, 1 µL SUPERaseIn [Promega], 0.5 µL TAP) at 37°C for 1.5 h, and then pre-incubated with PNK reaction premix (1 µL PNK [NEB], 1 µL 300 mM MgCl_2_, 1 µL 100 mM ATP) for 30 min. Afterward, PNK reaction main mix (20 µL PNK buffer [NEB], 2 µL 100 mM ATP [Roche], and 142 µL water, 1 µL SUPERaseIN [Promega], and another 2 µL PNK [NEB]) was added to the pre-incubated RNA sample and incubated at 37°C for 30 min. The RNA was extracted and precipitated again as above and resuspended in 9 µL H_2_O.

#### 5′ adapter ligation

BrU-RNA, 5′ adapter (5 µM), and PEG were heated at 65°C for 5 min then cooled on ice. Ligation mixture (1.5 µL 5′ adapter [5 µM], 2 µL 10× RNA ligation buffer, 1.5 µL T4 RNA ligase, 1 µL SUPERaseIn, 5 µL 50% PEG 8000) was added to the 9 µL BrU-RNA and incubated at 22°C or RT for 4–6 h. Then, 5′ adapter-ligated BrU-RNA was purified with the bead binding method as described above.

#### 3′ adapter ligation

The same ligation reaction for the 5′ adapter ligation described above was performed with the 3′ adapter in place of the 5′ adapter.

#### RT reaction

RNA and RT oligo (5′-CAAGCAGAAGACGGCATACGA-3′) were heated to 65°C for 10 min and cooled on ice. RT reagent mixture (1 µL RT oligo [100 µM], 5× first strand buffer [Invitrogen], 10 mM dNTPs [Roche], 100 mM DTT [Invitrogen], 1 µL RNase inhibitor [Promega] without Superscript III [Invitrogen]) was added to the RNA sample and incubated at 48°C for 3 min, and then 1 µL Superscript III was added to the RT reaction sample and incubated at 48°C for 20 min and 50°C for 45 min, sequentially. After the RT reaction, RNA was eliminated by adding RNase cocktail and RNase H and incubating at 37°C for 30 min.

#### PCR amplification

The ssDNA template was amplified by PCR using the Phusion High-Fidelity enzyme (NEB) according to the manufacturer's instructions. The small RNA PCR primers (5′-CAAGCAGAAGACGGCATACGA-3′ and 5′-AATGATACGGCGACCACCGACAGGTT-3′) were used to generate DNA for sequencing. PCR was performed with an initial 5-min de-naturation at 98°C, followed by 10∼14 cycles of 10-sec denaturation at 98°C, 30-sec annealing at 54°C, and 15-sec extension at 72°C. The PCR product was purified by running on a 6% native polyacrylamide TBE gel and recovered by cutting the region of the gel between 100 bp and 300 bp. The product was purified through the gel extraction method. The prepared DNA was then sequenced on the Illumina Genome Analyzer II according to the manufacturer's instructions with small RNA sequencing primer 5′-CGACAGGTTCAGAGTTCTACAGTCCGACGATC-3′.

### eRNA annotation

GRO-seq reads were mapped to human genome assembly hg18 using Bowtie 2 (Langmead and Salzberg 2012). We merged GRO-seq data across time points and used HOMER (Heinz et al. 2010) for de novo transcript identification with option “-style groseq”. Intergenic transcripts, which were >1 kb from 5′ ends and >10 kb from 3′ ends of RefSeq gene annotations, were selected as eRNA candidates. The RefSeq annotation was downloaded through an R package called “GenomicFeatures” (Lawrence et al. 2013), with “GenomicFeature” version 1.20.3 and creation time “2015-11-24 13:48:33 -0600 (Tues., Nov. 24, 2015)”. We filtered out regions that did not overlap with either H3K4me1 or H3K27ac peak regions.

ENCODE epigenetic data analyzed here can be downloaded from GEO under accession numbers GSE29611 (H3K4me1 and H3K27ac), GSE29692 (DNase-seq), GSE35586 (MNase-seq), and GSE31477 (EP300). Human enhancer atlas data were downloaded from http://slidebase.binf.ku.dk/human_enhancers/, the permissive enhancer set.

### Expression analysis of coding and noncoding transcription

Expression levels of genes and enhancers were calculated as reads per kilobase per million (RPKM). R package DESeq2 (Love et al. 2014) was used to perform differential analysis between two time points. Differential gene sets were submitted to David Bioinformatics Resources Database (Huang da et al. 2009a,b) for functional enrichment analysis. Principle component analysis and t-distributed stochastic neighbor embedding methods were applied with expressed genes/enhancers (mean RPKM > 0.5) for data visualization.

We designed one-step delayed auto-correlation to control noise levels and the absolute fold change to identify responsive genes/enhancers. Selected genes/enhancers were subjected to clustering by the “Partitioning Around Medoids” (PAM) algorithm, resulting in three clusters: “inducible early,” “inducible late,” and “repressed.”

### Determining inducible enhancers and genes

We identified inducible enhancers/genes using the amplitude index and continuity index. The expression level at time *t* is represented as *e*(*t*). *AI* is defined as the maximum logarithm fold increases before 24 h,

$$AI=\log_{2}\;[\frac{\underset{t\leq 24\;h}{\max}e(t)}{e(0)}].$$

*CI* is defined as the one-step delayed auto-correlation, to filter out enhancers/genes with noisy expression pattern

$$CI=\mathrm{correlation}\{[e(t_{1}),\ldots ,e(t_{n-1})],[e(t_{2}),\ldots ,e(t_{n})]\}.$$

We then selected enhancers and genes with *AI* > 1 and *CI* > 0.2 as inducible.

### Concordant and discordant EP pairs

Inducible EP pairs were ranked by the Spearman's correlation coefficients (SCCs) between enhancers and genes. Pairs ranked at the top 30% and bottom 30% of the list are designated as concordant and discordant, respectively.

### Pairing enhancer and target genes

Fold changes at each time point were calculated for enhancers near inducible genes. We divided enhancers into groups according to their distance from genes and found enhancers <200 kb from these genes showed significantly stronger inducibility. We named inducible genes and enhancers within 200 kb distance as inducible EP pairs.

### Motif analysis

We used TF binding motif PWM matrices from HOmo sapiens COmprehensive MOdel COllection (HOCOMOCO) v10 (Kulakovskiy et al. 2016). We applied HOMER module annotatePeaks.pl to identify motif occurrence in inducible enhancers and genes (see scripts in Supplemental_Script_S1).

### Synteny analysis

We analyzed EP colocalization in 11 species covering different levels of metazoan animals, including chimp, marmoset, mouse, rat, guinea pig, rabbit, cow, dog, elephant, armadillo, and lizard. Orthologs of enhancers and promoters were identified using the UCSC LiftOver tool with minimal match ratio set to 0.1. We tested the percentage of inducible human EP pairs locating in the same chromosome in other species. For statistical analysis, we generated a background set by paring 10,000 random promoter regions with the same number of intergenic regions, following the distance distribution of inducible pairs.

### Chromatin immunoprecipitation (ChIP) sequencing

Chromatin was prepared and immunoprecipitated as described previously (Kim et al. 2011), except that protein A/G Dynabeads (Invitrogen) were used instead of organism-specific secondary antibody bound beads. Twenty-five percent of the amount of chromatin was used to reduce oversaturation of bead binding capacity. H3K27ac antibody from Abcam (ab4729) was used for the ChIP experiment. The ThruPLEX DNA-seq kit from Rubicon Genomics was used for multiplexed ChIP-seq and input sample library prep of GM12878 chromatin. Indexed samples were quantitated with qPCR and mixed in equimolar amounts. The Yale Stem Cell Center Genomics and Bioinformatics Core Facility conducted the sequencing on an Illumina HiSeq 2000 platform. ChIP-seq peaks were called with MACS2 (Zhang et al. 2008) with the default mode. We analyzed our ChIP-seq data using deepTools2 (Ramirez et al. 2016) and customized scripts (see scripts in Supplemental_Script_S2).

### Chromosome conformation capture (3C)

The 3C assay was performed as described (Kim et al. 2011; Banerjee et al. 2014), with minor modifications. Briefly, one million cells were crosslinked with 1% formaldehyde for 15 min at room temperature resuspended in lysis buffer (10 mM Tris-Cl at pH 8.0, 10 mM NaCl, and 0.2% NP-40), and incubated on ice for 90 min. Ten million of these prepared nuclei were digested with EcoRI (New England Biolabs) overnight at 37°C, followed by ligation with T4 DNA ligase (New England Biolabs) at 16°C for 4 h. The ligated DNA was incubated with Proteinase K at 65°C for 12 h to reverse the crosslinks. Following incubation, the DNA was treated with RNase A. The treated DNA was extracted with phenol:chloroform and precipitated with sodium acetate and ethanol. The DNA concentration of the recovered 3C library was determined using a Qubit dsDNA HS assay kit (Invitrogen). Quantitative real-time PCR was performed to confirm the specific ligation between two DNA fragments in the sample and control 3C libraries. The position and sequence of primers designed for the 3C qPCR assay are listed in Supplemental Table S4. Interaction frequencies were calculated by dividing the amount of PCR product obtained with the sample 3C library constructed from nuclei by the amount of PCR product obtained with the control library DNA generated from ligating EcoRI fragments from the corresponding bacterial artificial clones (BAC) (Supplemental Table S4): interaction $\mathrm{frequency}=2^{\left(\Delta C_{t}\;\mathrm{sample}-\Delta C_{t}\;\mathrm{control}\right)}$. All 3C analyses were performed, at a minimum, in triplicate.

### eRNA KD analysis with siRNA

siRNA duplex for eRNA KD was obtained from Sigma-Aldrich. Their sequences and eRNA region of induced EP pairs and control EP pairs are listed in Supplemental Tables S5 and S6, respectively. As a negative control, scrambled siRNA was used. As a mock control, only transfection reagent without siRNA was added to the cell sample. Three hundred thousand cells were prepared in 800 µL media in each well of a 12-well plate. Separately, siRNA transfection solution was prepared by adding 1 µL of siRNA (10 µM stock of siRNA) and 5 µL of Mission siRNA transfection reagent (Sigma) to 200 µL OPTI-MEM, followed by incubation for 15–20 min at room temperature. Then, siRNA transfection solution was added to the cells carefully, by dropping it, and incubated for 5 h at 37°C, and then changed with fresh media. After 36 h of incubation, virus solution with the concentration of 50 µL/mL media was added to the cells to activate the inducible immune response gene system. After 12 h, total RNA was extracted with adding 500 µL of TRIzol solution (Invitrogen) to the cell pellet spun down at 1500 rpm for 3 min and rotated at 4°C for 5 min.

RT-qPCR was performed to check the transcription level after siRNA KD for eRNA and promoter RNA. Total RNA extract with TRIzol (Invitrogen) was treated with DNase I (Roche) for 30 min at 37°C and further extracted with acidic phenol:chloroform and precipitated with salt, glycogen, and pure ethanol. The RNA was reverse-transcribed using ImProm-II (Promega) with 100 µM of oligo(dT) primers or random decamers. The resulting cDNA was incubated with 10 µg of RNase H and RNase cocktail for 30 min at 37°C and purified using a PCR purification kit (MACHEREY-NAGEL). Five to 10 ng of purified cDNA was quantified by using a FastStart Universal SYBR Green Master Mix (Roche) on a qPCR machine (Realplex2, Eppendorf). We used *GAPDH* as the internal control. The *GAPDH* primers for RT-qPCR are: forward 5′-TGCACCACCAACTGCTTAGC-3′ and reverse 5′-GGCATGGACTGTGGTCATGAG-3′. To calculate the relative expression fold change (sample/control), we used the scrambled siRNA transfection as the negative control. The qPCR primers were designed against each siRNA-targeting region of eRNA and promoter, and the sequences of primers were listed in Supplemental Tables S8 and S9.

### Apoptosis assay

To evaluate cell viability, we performed western blots with cleaved caspase 3 antibody (Cell Signaling Technology, #9661) and an Annexin-V (ANXA5) apoptosis detection flow cytometry assay as described (Goodwin et al. 2017). Cells were infected at 36 h after siRNA transfection as described in the previous Methods section (targeting *TNFSF10* eRNAs and *IFNB1* eRNA, *L2*), and cells (300,000 per well) were harvested at 24, 72, and 96 h after virus infection with cold PBS wash. For the negative control experiment, 5 µM of TIC10 (SML1068, Sigma-Aldrich) were treated for 48 h to activate the apoptosis pathway by inducing the level of *TNFSF10* expression.

For the western blot, in order to extract protein from each well, 40 µL of RIPA buffer with freshly made proteinase inhibitor cocktail (Roche) was added to the cell pellet. A 12% SDS gel was run for 1 h with constant voltage (120 V), followed by transfer to a membrane (Immun-Blot PVDF membrane sandwiches, Bio-Rad) with constant 0.1 A for 45 min. The size of cleaved caspase 3 is 17–19 kDa. β-Actin (45 kDa) was used as an internal standard.

For flow cytometry, cell death was measured using the PE Annexin-V Apoptosis Detection Kit I (BD Pharmingen) according to the manufacturer's instructions. Cells were collected and stained with annexin-V and 7-AAD and analyzed by flow cytometry (SH800, Sony) and FlowJo software.

## Data access

GRO-seq and ChIP-seq data from this study have been submitted to the Array Express (http://www.ebi.ac.uk/arrayexpress/) under accession numbers E-MTAB-6047 and E-MTAB-6050.

## Supplementary Material

Supplemental Material
