# Supplementary material for: Global transcriptional activity dynamics reveal functional enhancer RNAs
Source: Genome Res. 2018 Dec;28(12):1799–811. doi: 10.1101/gr.233486.117 (PMC6280751; doi:10.1101/gr.233486.117)
Supplement: Supplemental Material [file supp_28_12_1799__index.html]

Global transcriptional activity dynamics reveal functional enhancer RNAs — Supplemental Material 

# Global transcriptional activity dynamics reveal functional enhancer RNAs

## Supplemental Material

- Supplemental\_Material.pdf
- Supplemental\_Script2.zip
- Supplemental\_Script1.zip
