# Supplementary material for: Global transcriptional activity dynamics reveal functional enhancer RNAs
Source: Genome Res. 2018 Dec;28(12):1799–811. doi: 10.1101/gr.233486.117 (PMC6280751; doi:10.1101/gr.233486.117)
Supplement: Supplemental Material [file supp_gr.233486.117_Supplemental_Material.pdf]

Supplemental Figures

Supplemental Figure 1. Differentially expressed genes and enhancer by SeV virus infection

(a) Histogram of eRNA lengths is shown. X-axis represents the $\log_{10}$ of eRNA lengths and Y-axis represents the frequency. **(b)** Gene Ontology terms enriched by up-regulated genes (UP) and down-regulated gene (DOWN) are shown with spans of arrows showing duration of gene activation. **(c)** and **(d)** Representative genome browser track views of activated immune-related genes, MX1, ISG15 and CCL5, and activated enhancers. Epigenetic (DNase HS, H3K4me1 or H3K4me3 and H3K27ac) signals were normalized to the maximum levels in the regions. The y-axis of GRO-seq data represents normalized read density in reads per 10 million. **(e)** and **(f)** The results from t-distributed stochastic neighbor embedding (t-SNE) dimension-reduction analysis for mRNA and eRNA are shown. The plots are color-coded with blue and orange representing early and late time points, respectively. **(g)** K27ac enrichment level at the inducible enhancer with time course after virus infection is shown.

Supplemental Figure S1

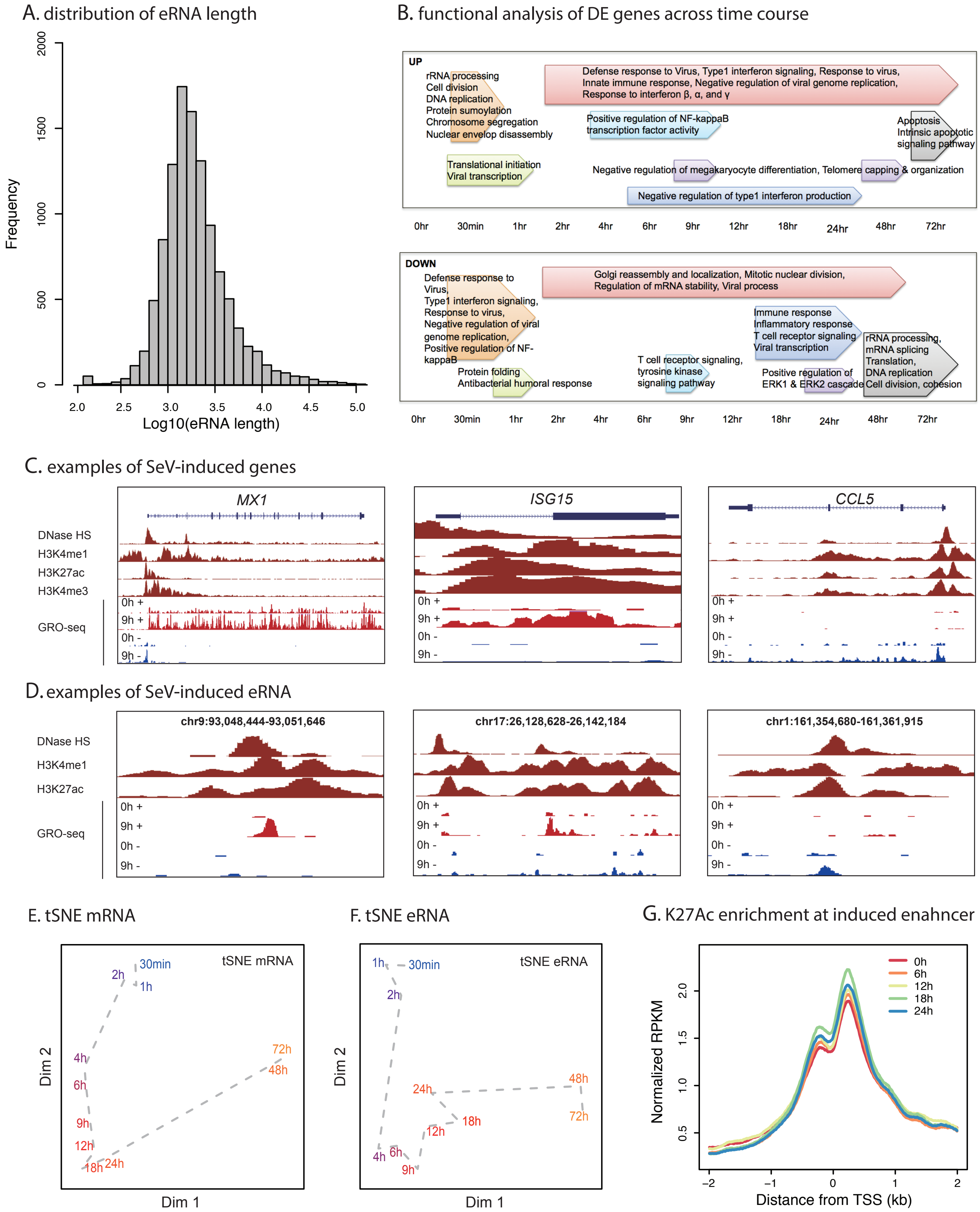

Supplemental Figure 2. *L2*, *IFNB1* enhancer and *IFNB1* transcriptional changes before (0h) and after virus infection (9h). (a) Genome browser track of *L2* and *IFNB1* is shown. Epigenetic (Sg1, H3K4me1/3 and H3K27ac) signals were normalized by the maximum levels in the regions. The Y-axis represents GRO-seq signal in reads per 10 million. **(b)** Heat map summarizes GRO-seq RPKM level of *L2* and *IFNB1* as well as its signal related genes, IRFs, NFκBs, and RELs through the incubation times from 0 hour to 24 hours after infection.

Supplemental Figure S2

A.

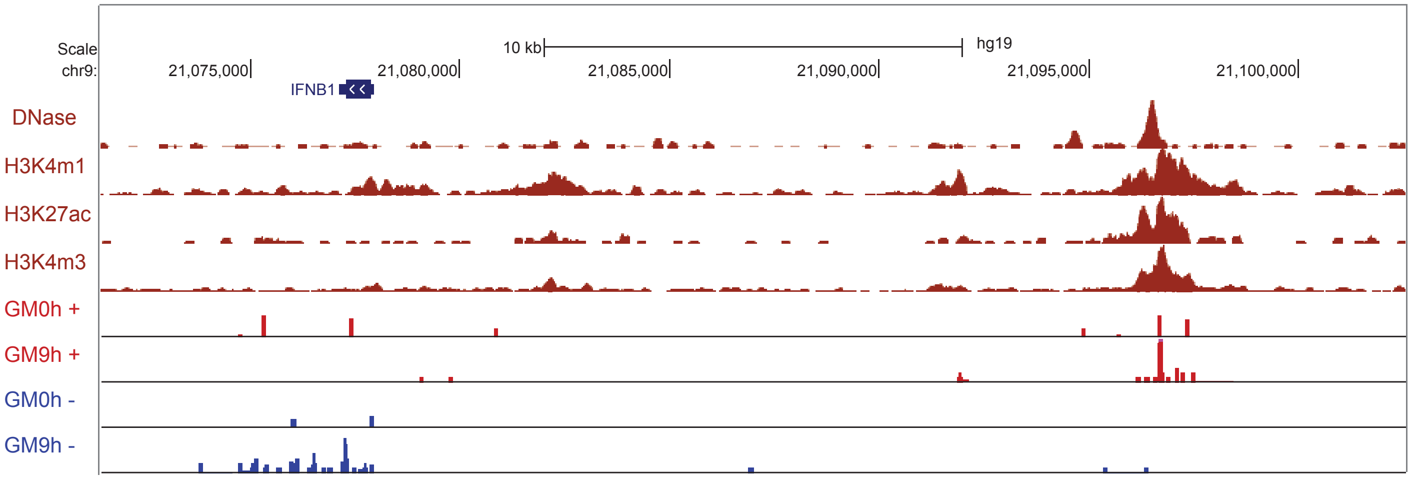

B.

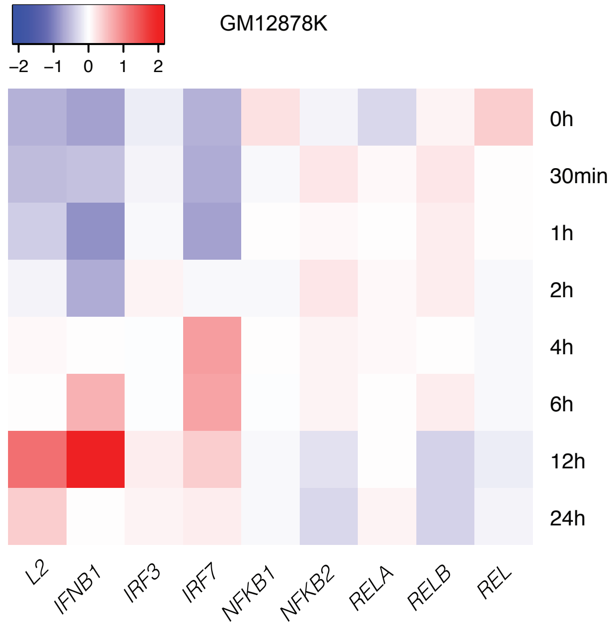

Supplemental Figure 3. Prediction of virus inducible enhancer-promoter (EP) pairs. (a) Average logarithm fold-changes of expressed enhancers near inducible genes, divided into two groups according to EP distance are shown: 0-100kb, 100-200kb, 200-300kb, 300-400kb, 400-500kb, and 500-600kb. **(b)** Distribution of EP distance between inducible (red) and background (grey) pairs is shown. **(c)** Average phastCon conservation scores of each mammal and vertebrate across inducible enhancer regions, relative to randomly selected background are shown. **(d)** Percentage of inducible human EP pairs (EP distance < 500kb) that co-exist in other species is shown. **(e)** Violin plots showing the logarithmic fold change values of enhancers at each time point after infection. Enhancers were divided into groups based on distances from inducible genes.

Supplemental Figure S3

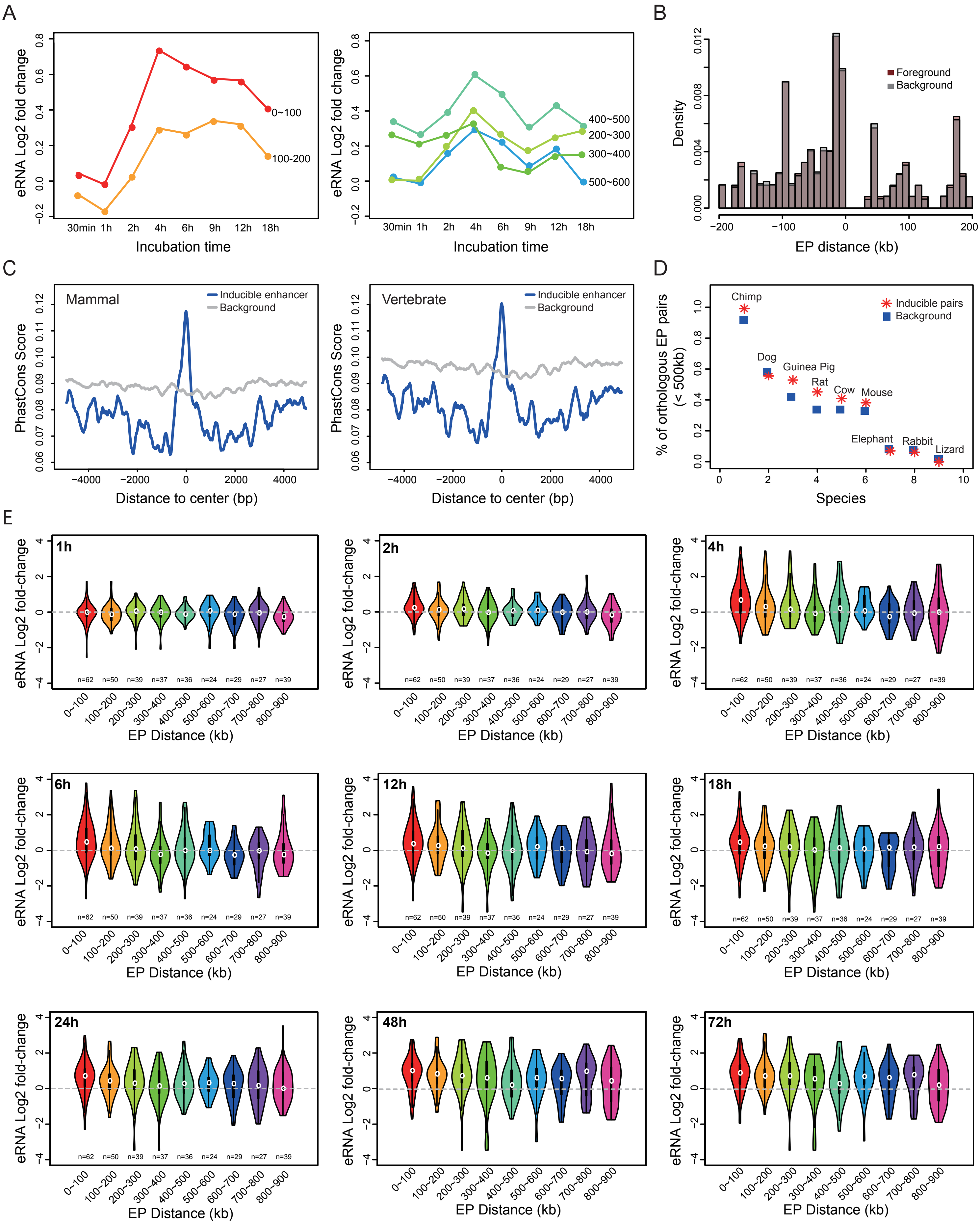

Supplemental Figure 4. Effects of eRNA KD on target gene transcription. Individual KD experiments of inducible EP pairs are shown. Barplots (left) show the ratio of eRNA (blue) / mRNA (orange) expression level before and after siRNA treatment. Expression profiles (right) show GRO-seq signal (RPKM) of eRNA (blue) / mRNA (orange) during virus incubation time.

Supplemental Figure S4

A eRNA knockdown experiments

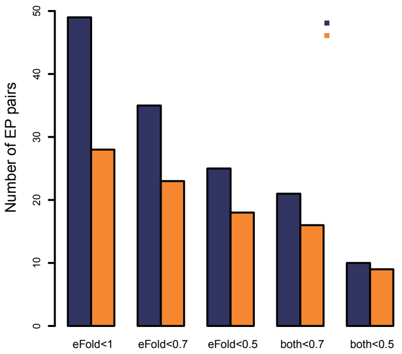

B

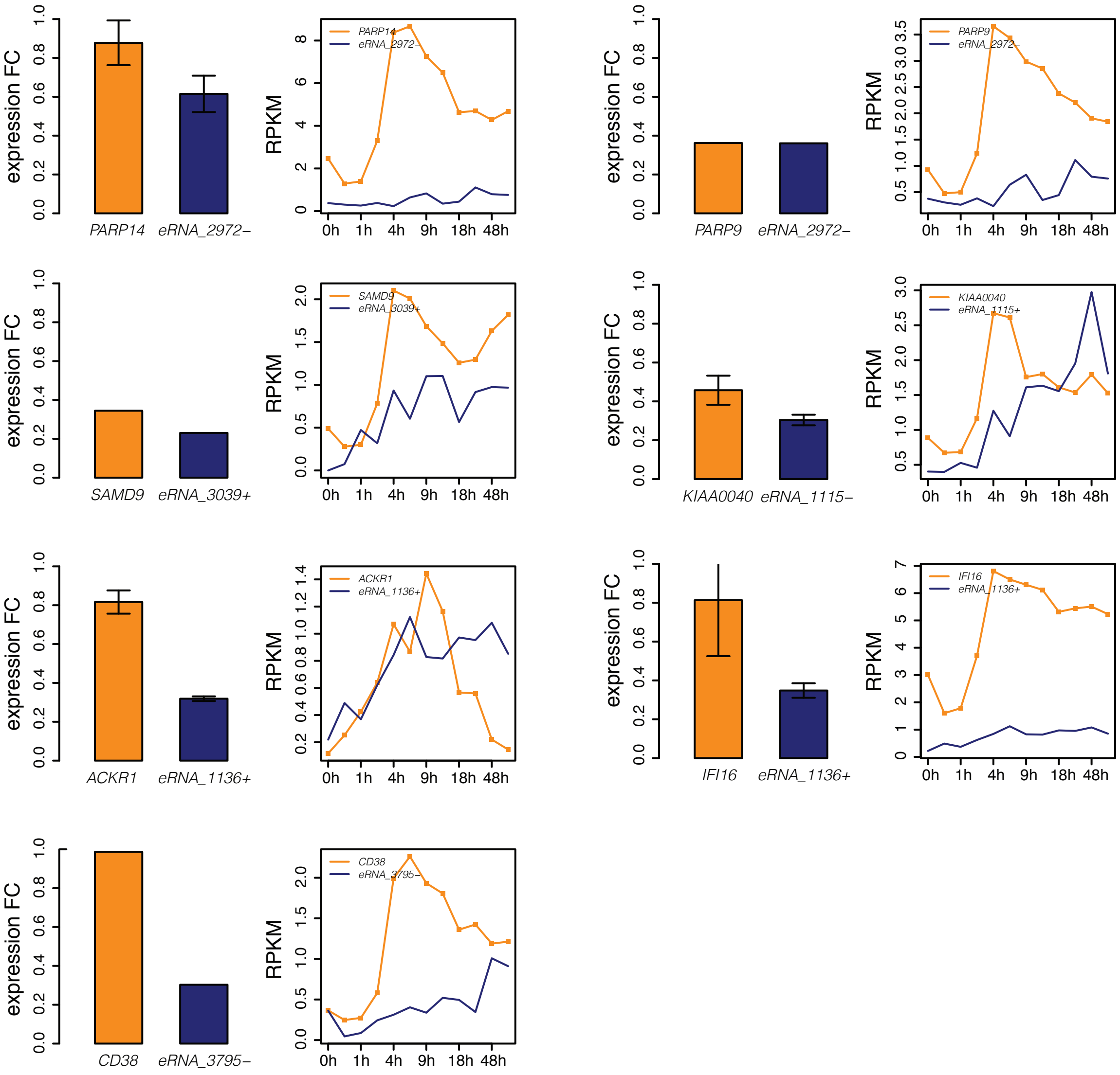

Supplemental Figure 5. eRNA KD effect on physical EP interaction. (a), (b), and(c) (Top) Schematic diagrams of *IFNB1*/*IFI35*/*MYCBP2* and corresponding enhancer are shown. (Lower left) Fold-change of 3C interaction between EPs after eRNA KD is shown, compared with negative controls.

Supplementary Figure 5

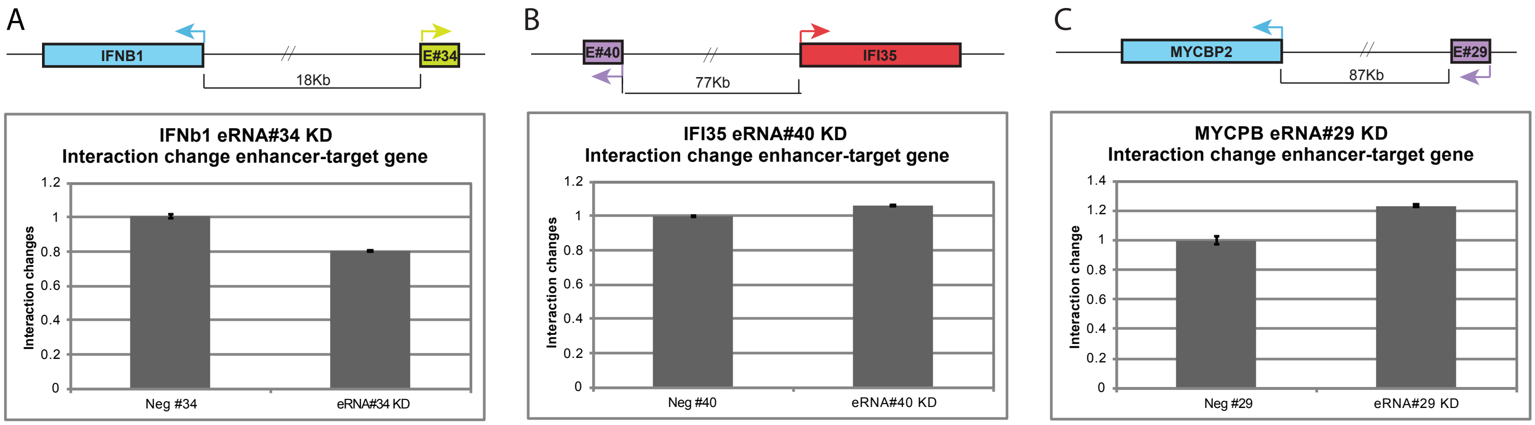

Supplemental Figure 6. Cases of genes with multiple enhancers. (a) and (b) Schematic diagram of *TLR7* and *CD38* and their nearby inducible enhancers is shown (top). Fold changes of eRNA (blue) / mRNA (orange) after individual and combined eRNA KD is plotted.

Supplementary Figure 6

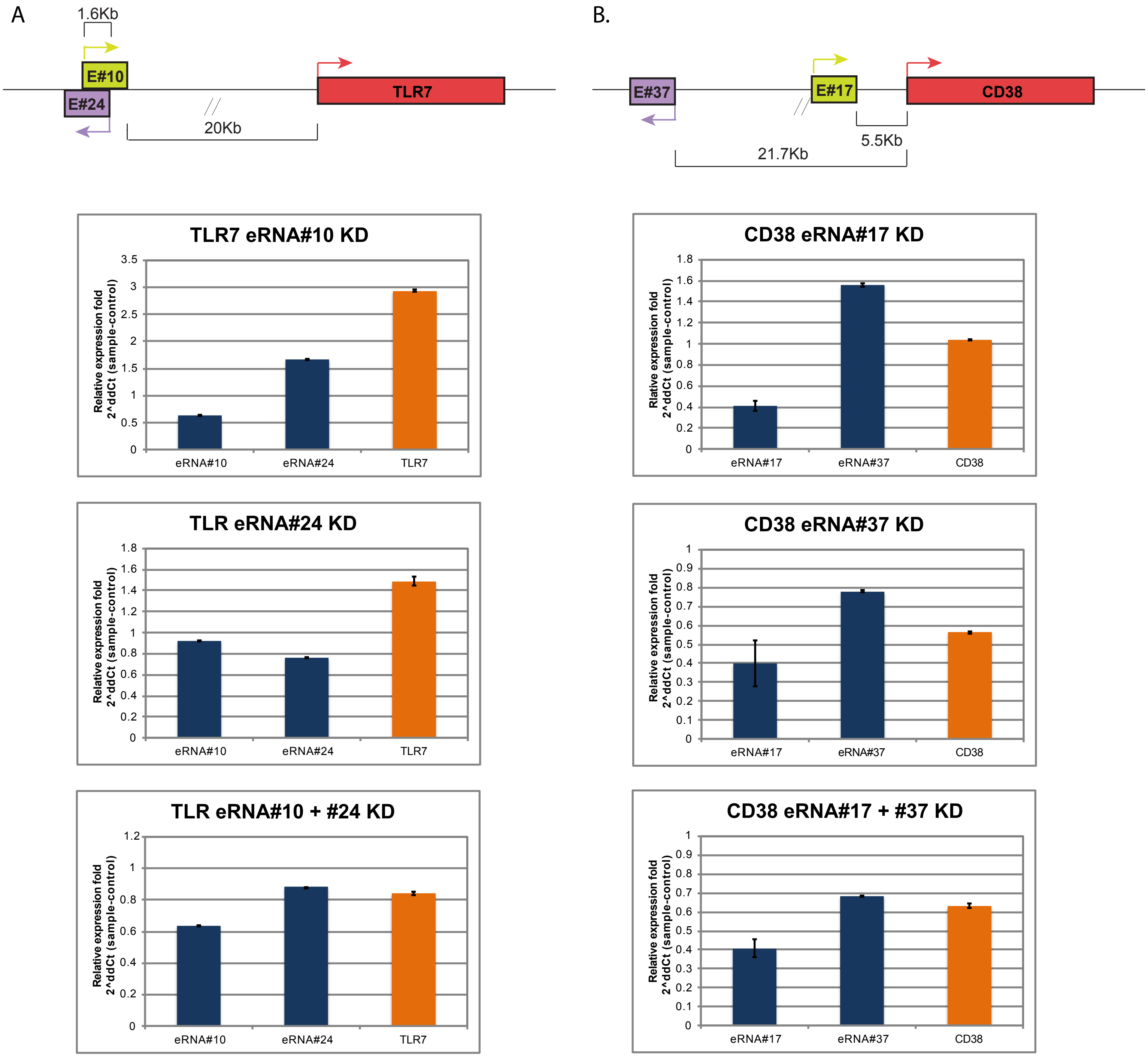

Supplemental Figure 7. eRNA KD and TIC10 treatment. Y-axis shows the fraction of apoptotic and necrotic cells after 48h TIC10 incubation, with single, triple or control eRNA KD.

Supplemental Figure S7

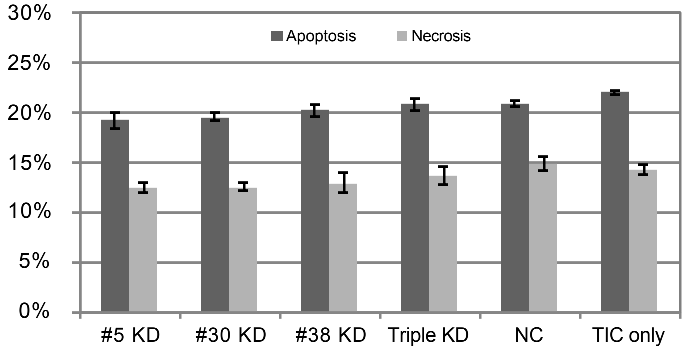

Supplemental Figure 8. Uncropped original view of the western blots in Fig 3C.

Supplemental Figure S8

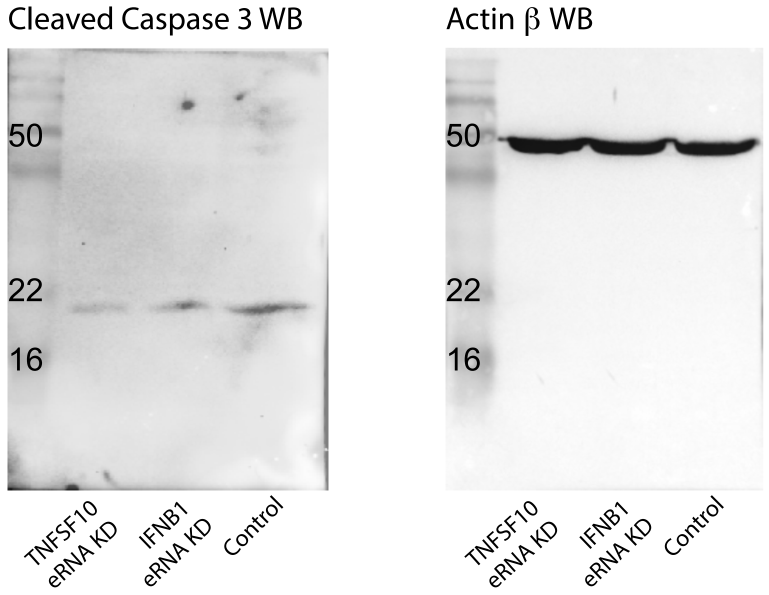

Supplemental Tables

Table S1. All predicted induced eRNAs and their target genes. Target_genes (Refseq) column notes accession number of target genes paired to inducible enhancer. Enhancer_id column contains unique IDs for inducible enhancers identified. Target-gene (symbol) column notes the gene name assigned to the Refseq accession number. Enhancer_coord_hg19 column refers to the genomic coordinate of the inducible enhancers based on the hg19 build of the human genome.

All induced EP pairs passing our criteria

Target_gene (Refseq)	Enhancer_id	Target_gene (symbol)	Enhancer_coord_hg19
NM_002036	*MetaEnhancer_1136_+*	*ACKR1*	chr1 158975648 158977353
NM_001122951	*MetaEnhancer_1136_+*	*ACKR1*	chr1 158975648 158977353
NM_004833	*MetaEnhancer_1136_+*	*AIM2*	chr1 158975648 158977353
NM_001136540	*MetaEnhancer_1799_-*	*APOL1*	chr22 36805195 36806192
NM_001136540	*MetaEnhancer_862_-*	*APOL1*	chr22 36846877 36848115
NM_030882	*MetaEnhancer_1799_-*	*APOL2*	chr22 36805195 36806192
NM_145637	*MetaEnhancer_1799_-*	*APOL2*	chr22 36805195 36806192
NM_030641	*MetaEnhancer_3137_-*	*APOL6*	chr22 36030413 36031205
NM_174919	*MetaEnhancer_2379_-*	*ARHGAP27*	chr17 43433743 43435137
NM_001128616	*MetaEnhancer_2422_+*	*ARHGEF3*	chr3 56726327 56729181
NM_019555	*MetaEnhancer_2422_+*	*ARHGEF3*	chr3 56726327 56729181
NM_001731	*MetaEnhancer_3101_+*	*BTG1*	chr12 92586863 92587704
NM_001295	*MetaEnhancer_258_-*	*CCR1*	chr3 46149958 46152633
NM_001295	*MetaEnhancer_1961_+*	*CCR1*	chr3 46150916 46156022
NM_001775	*MetaEnhancer_3795_-*	*CD38*	chr4 15757373 15758209
NM_001775	*MetaEnhancer_847_-*	*CD38*	chr4 15767579 15773083
NM_001775	*MetaEnhancer_1694_+*	*CD38*	chr4 15773236 15774381
NM_001243794	*MetaEnhancer_2191_+*	*CHST12*	chr7 2439830 2441170
NM_172210	*MetaEnhancer_3084_+*	*CSF1*	chr1 110435830 110436993
NM_172212	*MetaEnhancer_3084_+*	*CSF1*	chr1 110435830 110436993
NM_000757	*MetaEnhancer_3084_+*	*CSF1*	chr1 110435830 110436993
NM_138287	*MetaEnhancer_2972_-*	*DTX3L*	chr3 122380136 122380883
NM_001034194	*MetaEnhancer_2326_-*	*EXOSC9*	chr4 122708957 122710268
NM_001034194	*MetaEnhancer_1507_-*	*EXOSC9*	chr4 122714858 122717023
NM_001034194	*MetaEnhancer_1473_+*	*EXOSC9*	chr4 122716718 122718546
NM_153230	*MetaEnhancer_2571_-*	*FBXO39*	chr17 6842901 6843791
NM_014824	*MetaEnhancer_94_+*	*FCHSD2*	chr11 72864171 72888511
NM_002053	*MetaEnhancer_1625_+*	*GBP1*	chr1 89546827 89547678
NM_052941	*MetaEnhancer_1625_+*	*GBP4*	chr1 89546827 89547678
NM_052941	*MetaEnhancer_2172_+*	*GBP4*	chr1 89739974 89742358
NM_001206567	*MetaEnhancer_1136_+*	*IFI16*	chr1 158975648 158977353
NM_002038	*MetaEnhancer_1613_-*	*IFI6*	chr1 28014868 28016573
NM_001270927	*MetaEnhancer_2029_-*	*IFIT1*	chr10 91052142 91055958
NM_001270927	*MetaEnhancer_3024_+*	*IFIT1*	chr10 91055858 91057638
NM_001547	*MetaEnhancer_4002_-*	*IFIT2*	chr10 90922933 90923624
NM_001547	*MetaEnhancer_1253_+*	*IFIT2*	chr10 90923524 90925010
NM_001547	*MetaEnhancer_2029_-*	*IFIT2*	chr10 91052142 91055958
NM_001547	*MetaEnhancer_3024_+*	*IFIT2*	chr10 91055858 91057638
NM_001289758	*MetaEnhancer_4002_-*	*IFIT3*	chr10 90922933 90923624
NM_001031683	*MetaEnhancer_4002_-*	*IFIT3*	chr10 90922933 90923624

NM_001289758	*MetaEnhancer_1253_+*	*IFIT3*	chr10 90923524 90925010
NM_001031683	*MetaEnhancer_1253_+*	*IFIT3*	chr10 90923524 90925010
NM_001289758	*MetaEnhancer_2029_-*	*IFIT3*	chr10 91052142 91055958
NM_001031683	*MetaEnhancer_2029_-*	*IFIT3*	chr10 91052142 91055958
NM_001289758	*MetaEnhancer_3024_+*	*IFIT3*	chr10 91055858 91057638
NM_001031683	*MetaEnhancer_3024_+*	*IFIT3*	chr10 91055858 91057638
NM_012420	*MetaEnhancer_2029_-*	*IFIT5*	chr10 91052142 91055958
NM_012420	*MetaEnhancer_3024_+*	*IFIT5*	chr10 91055858 91057638
NM_003641	*MetaEnhancer_1268_+*	*IFITM1*	chr11 188083 189410
NM_003641	*MetaEnhancer_1341_-*	*IFITM1*	chr11 347562 351028
NM_003641	*MetaEnhancer_230_-*	*IFITM1*	chr11 353806 356185
NM_006435	*MetaEnhancer_1268_+*	*IFITM2*	chr11 188083 189410
NM_006435	*MetaEnhancer_1341_-*	*IFITM2*	chr11 347562 351028
NM_006435	*MetaEnhancer_230_-*	*IFITM2*	chr11 353806 356185
NM_002176	*MetaEnhancer_1571_+*	*IFNB1*	chr9 21096515 21098279
NM_002198	*MetaEnhancer_250_-*	*IRF1*	chr5 131828856 131832505
NM_002198	*MetaEnhancer_372_+*	*IRF1*	chr5 131831379 131847154
NM_002163	*MetaEnhancer_2143_+*	*IRF8*	chr16 85922550 85923645
NM_002163	*MetaEnhancer_259_+*	*IRF8*	chr16 86028925 86034443
NM_005547	*MetaEnhancer_3423_+*	*IVL*	chr1 152844459 152845369
NM_005547	*MetaEnhancer_2511_+*	*IVL*	chr1 152940836 152941593
NM_001162895	*MetaEnhancer_633_-*	*KIAA0040*	chr1 175173105 175173615
NM_001162893	*MetaEnhancer_633_-*	*KIAA0040*	chr1 175173105 175173615
NM_001162895	*MetaEnhancer_1625_-*	*KIAA0040*	chr1 175201951 175206691
NM_001162893	*MetaEnhancer_1625_-*	*KIAA0040*	chr1 175201951 175206691
NM_001162895	*MetaEnhancer_3581_-*	*KIAA0040*	chr1 175255261 175256747
NM_001162893	*MetaEnhancer_3581_-*	*KIAA0040*	chr1 175255261 175256747
NM_001162895	*MetaEnhancer_1115_+*	*KIAA0040*	chr1 175256708 175258040
NM_001162893	*MetaEnhancer_1115_+*	*KIAA0040*	chr1 175256708 175258040
NM_002308	*MetaEnhancer_606_-*	*LGALS9*	chr17 26131567 26137792
NM_002308	*MetaEnhancer_211_+*	*LGALS9*	chr17 26135260 26137784
NM_002308	*MetaEnhancer_421_+*	*LGALS9*	chr17 26137777 26140602
NM_002432	*MetaEnhancer_1136_+*	*MNDA*	chr1 158975648 158977353
NM_013262	*MetaEnhancer_3166_+*	*MYLIP*	chr6 15989743 15990881
NM_022750	*MetaEnhancer_1844_+*	*PARP12*	chr7 139910100 139913318
NM_017554	*MetaEnhancer_2972_-*	*PARP14*	chr3 122380136 122380883
NM_001146102	*MetaEnhancer_2972_-*	*PARP9*	chr3 122380136 122380883
NM_001146104	*MetaEnhancer_2972_-*	*PARP9*	chr3 122380136 122380883
NM_001146103	*MetaEnhancer_2972_-*	*PARP9*	chr3 122380136 122380883
NM_001146106	*MetaEnhancer_2972_-*	*PARP9*	chr3 122380136 122380883
NM_021105	*MetaEnhancer_4023_-*	*PLSCR1*	chr3 146268426 146269080
NM_002787	*MetaEnhancer_1544_-*	*PSMA2*	chr7 43110543 43112396
NM_006871	*MetaEnhancer_844_-*	*RIPK3*	chr14 24741839 24743689

NM_001193307	*MetaEnhancer_3039_+*	*SAMD9*	chr7 92666321 92666833
NM_001303496	*MetaEnhancer_3039_+*	*SAMD9L*	chr7 92666321 92666833
NM_015474	*MetaEnhancer_1375_-*	*SAMHD1*	chr20 35493353 35495639
NM_015295	*MetaEnhancer_510_-*	*SMCHD1*	chr18 2634970 2637532
NM_015295	*MetaEnhancer_730_-*	*SMCHD1*	chr18 2637665 2641759
NM_013306	*MetaEnhancer_2509_+*	*SNX15*	chr11 64918212 64918934
NM_001308205	*MetaEnhancer_2826_+*	*SSR3*	chr3 156323933 156325284
NM_001308204	*MetaEnhancer_2826_+*	*SSR3*	chr3 156323933 156325284
NM_001308197	*MetaEnhancer_2826_+*	*SSR3*	chr3 156323933 156325284
NM_001308205	*MetaEnhancer_2114_+*	*SSR3*	chr3 156361733 156363160
NM_001308204	*MetaEnhancer_2114_+*	*SSR3*	chr3 156361733 156363160
NM_001308197	*MetaEnhancer_2114_+*	*SSR3*	chr3 156361733 156363160
NM_001308205	*MetaEnhancer_3846_+*	*SSR3*	chr3 156374076 156375609
NM_001308204	*MetaEnhancer_3846_+*	*SSR3*	chr3 156374076 156375609
NM_001308197	*MetaEnhancer_3846_+*	*SSR3*	chr3 156374076 156375609
NM_016562	*MetaEnhancer_341_-*	*TLR7*	chrX 12859229 12863555
NM_016562	*MetaEnhancer_502_+*	*TLR7*	chrX 12862239 12865018
NM_001190942	*MetaEnhancer_2005_+*	*TNFSF10*	chr3 172194931 172195631
NM_001190943	*MetaEnhancer_2005_+*	*TNFSF10*	chr3 172194931 172195631
NM_001190942	*MetaEnhancer_3924_-*	*TNFSF10*	chr3 172263822 172264784
NM_001190943	*MetaEnhancer_3924_-*	*TNFSF10*	chr3 172263822 172264784
NM_001190942	*MetaEnhancer_3404_-*	*TNFSF10*	chr3 172295508 172299753
NM_001190943	*MetaEnhancer_3404_-*	*TNFSF10*	chr3 172295508 172299753
NM_001190942	*MetaEnhancer_734_-*	*TNFSF10*	chr3 172308346 172310066
NM_001190943	*MetaEnhancer_734_-*	*TNFSF10*	chr3 172308346 172310066
NM_001190942	*MetaEnhancer_116_+*	*TNFSF10*	chr3 172310235 172315325
NM_001190943	*MetaEnhancer_116_+*	*TNFSF10*	chr3 172310235 172315325
NM_001190942	*MetaEnhancer_811_-*	*TNFSF10*	chr3 172310301 172313961
NM_001190943	*MetaEnhancer_811_-*	*TNFSF10*	chr3 172310301 172313961
NM_025195	*MetaEnhancer_733_-*	*TRIB1*	chr8 126614669 126617093
NM_001282985	*MetaEnhancer_733_-*	*TRIB1*	chr8 126614669 126617093
NM_025195	*MetaEnhancer_1012_+*	*TRIB1*	chr8 126617345 126619945
NM_001282985	*MetaEnhancer_1012_+*	*TRIB1*	chr8 126617345 126619945
NM_030961	*MetaEnhancer_931_+*	*TRIM56*	chr7 100893754 100895706
NM_001301144	*MetaEnhancer_501_-*	*TRIM69*	chr15 45073139 45076695
NM_001301144	*MetaEnhancer_1609_+*	*TRIM69*	chr15 45076646 45078317
NM_020830	*MetaEnhancer_512_-*	*WDFY1*	chr2 224812655 224817461
NM_017523	*MetaEnhancer_2571_-*	*XAF1*	chr17 6842901 6843791
NM_001160417	*MetaEnhancer_1451_+*	*ZBP1*	chr20 56008141 56009316
NM_001160419	*MetaEnhancer_1451_+*	*ZBP1*	chr20 56008141 56009316

Table S2. Genomic information for eRNAs and target genes of induced EP pairs analyzed by 3C and siRNA knockdown experiments. Enhancer_ID column contains unique IDs (from Table S1) for annotated eRNAs and corresponding strand information. Whole region column contains genomic coordinates for the full eRNA transcript that was detected across the entire time course. High enrichment region column contains genomic coordinates of a region within eRNAs with high GRO-seq signal that was used for designing the siRNAs. eRNA length column denotes the length of the annotated eRNA encompassing the whole region of the annotated eRNAs. Target Genes column contains the gene name and DNA strand information for the assigned target genes. Whole region column contains genomic coordinate information of the target genes. First exon region columns contains coordinate of first exon region. Gene length column notes target gene length. All genomic coordinates and names are based on the hg19 build of the human genome.

eRNA and target genes of induced EP pairs

eRNA		Whole region			High enrichment region			eRNA length	Target Genes		Whole region			First exon region			Gene length
Enhancer_ID	strand	chr	start	end	chr	start	end		Gene name	strand	chr	start	end	chr	start	end	
MetaEnhancer_1136_+	+	chr1	157242272	157243977	chr1	157242460	157242561	1705	*ACKR1*	+	chr1	157440426	157442914	chr1	157440426	157441394	2488
MetaEnhancer_258_-	-	chr3	46124962	46127637	chr3	46127469	46127636	2675	*CCR1*	-	chr3	46218203	46224836	chr3	46224776	46224836	6633
MetaEnhancer_1694_+	+	chr4	15382334	15383479	chr4	15382463	15382581	1145	*CD38-1*	+	chr4	15389028	15459804	chr4	15389028	15389368	70776
MetaEnhancer_3795_-	-	chr4	15366471	15367307	chr4	15366966	15367155	836	*CD38-2*	+	chr4	15389028	15459804	chr4	15389028	15389368	70776
MetaEnhancer_1136_+	+	chr1	157242272	157243977	chr1	157242460	157242561	1705	*IFI16*	+	chr1	157246305	157291569	chr1	157246305	157246575	45264
MetaEnhancer_1571_+	+	chr9	21086515	21088279	chr9	21086643	21086794	1764	*IFNB1*	-	chr9	21067103	21067943	chr9	21067103	21067943	840
MetaEnhancer_259_+	+	chr16	84586426	84591944	chr16	84586456	84586609	5518	*IRF8*	+	chr16	84490274	84513712	chr16	84490274	84490330	23438
MetaEnhancer_1115_+	+	chr1	173523331	173524663	chr1	173523395	173523541	1332	*KIAA0040*	-	chr1	173392745	173428852	chr1	173428618	173428852	36107
MetaEnhancer_1136_+	+	chr1	157242272	157243977	chr1	157242460	157242561	1705	*MNDA*	+	chr1	157067791	157085894	chr1	157067791	157067971	18103
MetaEnhancer_2972_-	-	chr3	123862826	123863573	chr3	123863236	123863394	747	*PARP14*	+	chr3	123882361	123932377	chr3	123882361	123882607	50016
MetaEnhancer_2972_-	-	chr3	123862826	123863573	chr3	123863233	123863404	747	*PARP9*	-	chr3	123729449	123766213	chr3	123765975	123766213	36764
MetaEnhancer_3039_+	+	chr7	92504257	92504769	chr7	92504452	92504536	512	*SAMD9*	-	chr7	92566761	92585272	chr7	92585111	92585272	18511
MetaEnhancer_502_+	+	chrX	12772160	12774939	chrX	12772218	12772389	2779	*TLR7-1*	+	chrX	12795122	12818401	chrX	12795122	12795163	23279
MetaEnhancer_341_-	-	chrX	12769150	12773476	chrX	12771691	12771969	4326	*TLR7-2*	+	chrX	12795122	12818401	chrX	12795122	12795163	23279
MetaEnhancer_116_+	+	chr3	173792929	173798019	chr3	173796865	173797019	5090	*TNFSF10-1*	-	chr3	173705991	173723991	chr3	173723736	173723991	18000
MetaEnhancer_734_-	-	chr3	173791040	173792760	chr3	173792248	173792364	1720	*TNFSF10-2*	-	chr3	173705991	173723991	chr3	173723736	173723991	18000
MetaEnhancer_2005_+	+	chr3	173677625	173678325	chr3	173677765	173677876	700	*TNFSF10-3*	-	chr3	173705991	173723991	chr3	173723736	173723991	18000
MetaEnhancer_512_-	-	chr2	224520899	224525705	chr2	224525490	224525639	4806	*WDFY1*	-	chr2	224448308	224518296	chr2	224518108	224518296	69988

Table S3. Genomic information for eRNAs and target genes of control EP pairs analyzed by 3C and siRNA knockdown experiments. Enhancer_ID column contains unique IDs (from Table S1) for annotated eRNAs and corresponding strand information. Whole region column contains genomic coordinates for the full eRNA transcript that was detected across the entire time course. High enrichment region column contains genomic coordinates of a region within eRNAs with high GRO-seq signal that was used for designing the siRNAs. eRNA length column denotes the length of the annotated eRNA encompassing the whole region of the annotated eRNAs. Target Genes column contains the gene name and DNA strand information for the assigned target genes. Whole region column contains genomic coordinate information of the target genes. First exon region columns contains coordinate of first exon region. Gene length column notes target gene length. All genomic coordinates and names are based on the hg19 build of the human genome.

eRNA and target gene of Control EP pairs

eRNA		Whole region			High enrichment region			eRNA length	Target Genes		Whole region			First exon region			Gene length
Enhancer ID	strand	chr	start	end	chr	start	end		Gene name	strand	chr	start	end	chr	start	end	
MetaEnhancer_600_+	+	chr22	37680218	37682221	chr22	37680446	37680578	2003	*APOBEC3B*	+	chr22	37708349	37718730	chr22	37708349	37708421	10381
MetaEnhancer_600_+	+	chr22	37680218	37682221	chr22	37680446	37680578	2003	*APOBEC3D-1*	+	chr22	37747063	37759202	chr22	37747063	37747487	12139
MetaEnhancer_94_-	-	chr22	37677998	37680141	chr22	37679910	37680116	2143	*APOBEC3D-2*	+	chr22	37747063	37759202	chr22	37747063	37747487	12139
MetaEnhancer_1529_+	+	chr6	47490687	47491589	chr6	47490697	47490886	902	*CD2AP*	+	chr6	47553483	47702955	chr6	47553483	47553943	149472
MetaEnhancer_699_+	+	chr5	118395014	118397954	chr5	118395699	118396002	2940	*DMXL1-1*	+	chr5	118434982	118612721	chr5	1.18E+08	1.18E+08	177739
MetaEnhancer_454_-	-	chr5	118393146	118396611	chr5	118396304	118396575	3465	*DMXL1-2*	+	chr5	118434982	118612721	chr5	1.18E+08	1.18E+08	177739
MetaEnhancer_475_+	+	chr6	33103702	33107329	chr6	33105577	33105889	3627	*HLA-DMA*	-	chr6	33024368	33028877	chr6	33028703	33028877	4509
MetaEnhancer_157_-	-	chr6	32546422	32549330	chr6	32548510	32548783	2908	*HLA-DRA*	+	chr6	32515596	32520802	chr6	32515596	32515787	5206
MetaEnhancer_157_-	-	chr6	32546422	32549330	chr6	32548510	32548783	2908	*HLA-DRB5*	-	chr6	32593131	32605984	chr6	32605879	32605984	12853
MetaEnhancer_820_-	-	chr17	38333528	38334970	chr17	38334705	38334946	1442	*IFI35*	+	chr17	38412267	38420002	chr17	38412267	38412511	7735
MetaEnhancer_311_+	+	chr17	34064382	34065788	chr17	34064375	34064679	1406	*MLLT6*	+	chr17	34115398	34139582	chr17	34115398	34115520	24184
MetaEnhancer_544_-	-	chr13	76886469	76887807	chr13	76887554	76887756	1338	*MYCBP2*	-	chr13	76516792	76799178	chr13	76798609	76799178	282386
MetaEnhancer_327_+	+	chr2	64303953	64307226	chr2	64303970	64304149	3273	*PELI1*	-	chr2	64173289	64225109	chr2	64224718	64225109	51820
MetaEnhancer_1477_-	-	chr16	11297248	11299746	chr16	11297809	11297902	2498	*SOCS1-1*	-	chr16	11255774	11257540	chr16	11257436	11257540	1766
MetaEnhancer_810_-	-	chr16	11308132	11312802	chr16	11312513	11312648	4670	*SOCS1-2*	-	chr16	11255774	11257540	chr16	11257436	11257540	1766
MetaEnhancer_1228_+	+	chr20	55489469	55491648	chr20	55489512	55489777	2179	*ZBP1*	-	chr20	55612307	55629038	chr20	55628723	55629038	16731
MetaEnhancer_804_+	+	chr18	58324203	58326493	chr18	58324896	58325080	2290	*ZCCHC2*	+	chr18	58341637	58396798	chr18	58341637	58342576	55161
MetaEnhancer_246_+	+	chr15	78096744	78104705	chr15	78100853	78101069	7961	*ZFAND6*	+	chr15	78138964	78217790	chr15	78138964	78139206	78826

Table S4. 3C assay primers and BAC clone for induced EP interaction (a) and control EP interaction (b). eRNA column contains Enhancer ID (from Table S2) and primer sequence from eRNA region for 3C assay (eRNA – primer). Target genes column contain gene name of the corresponding target genes and primer sequences for promoter region (Target promoter – primer 1 and Target promoter – primer 2). Control column contains primer sequence from control region used for 3C assay. BAC clone column contains clone identifier for BAC clones used for 3C assay controls.

3C assay primers and BAC clone

A. Induced EP interaction

eRNA		Target genes			Control	BAC clone
Enhancer ID	eRNA - primer	Target gene name	Target promoter - primer1	Target promoter - primer2	Control primer	
MetaEnhancer_1136_+	TCACGAACACCCAGAGATGT	*ACKR1*	AACTCTGATGGCCTCCTCTG	TTGGTCACCCTTTCTCCAGG	TCCGTAGTGAAAGTTTTGGGA	RP11-621D16 +RP11-1065J8
MetaEnhancer_258_-	ACCACCAGACATTAGCCCAG	*CCR1*	GGAGGGCAGTGTTGTTCAAA	CTTCCTCACGGCATTGCTAC	CTGCTCTTGTTCTCCACTGC	RP11-793E15
MetaEnhancer_1694_+	TGCTGCCACAAGAACATTTG	*CD38-1*	TCCTGTTGTGTACCTGGCTT	GGAGTCCAAAGGCAGTCTCT	TGATCCTTTCCTTGGCCTCA	RP11-640L4
MetaEnhancer_3795_-	GTGTGTGTGTGTGTGTGTGT	*CD38-2*	GGAGACCCAGGGAAGAGTTG	TCCTGTTGTGTACCTGGCTT	ATTACAGCACTTTGGGAGGC	RP11-640L4
MetaEnhancer_1136_+	TCACGAACACCCAGAGATGT	*IFI16*	GCAGTGATCAAAATTATGTCCCA	TCCAAGGCCATCTACAGAGC	GTGCTGTCTCCCTTCTGTCT	RP11-265E4
MetaEnhancer_1571_+	TCCCCAGTCATTAGCACAGT	*IFNb1*	GCAAAGGAAAGCAAACGACC	TCCCCACTGCCTTGTTCATA	TCAGGTAATGTGATGCCTCCA	RP11-372B20
MetaEnhancer_259_+	ATAGCCTGCCCCAAATCACT	*IRF8*	GAAGTGCTCTGCTTTCCGAG	TGGGCATTTGGTGGAATTCg	TGTTAATTACCTGAAGCGCGT	RP11-478M13+RP11-152O13
MetaEnhancer_1115_+	TCTTGGGACCGTGAAAGTGT	*KIAA0040*	ATCCAAGTGTTTCCAACCGC	CTTTCTCCGAACGCTCAAGG	AAAGAAAATGCCAGGCCCAG	RP11-661N21
MetaEnhancer_1136_+	TCACGAACACCCAGAGATGT	*MNDA*	CAAATCAACGGGAGCAAGCT	ATCATGAGAACAGCACGGGA	TGAGTTGGCTGTAAATGTGTGT	RP11-110D10 + RP11-1065J8
MetaEnhancer_2972_-	TGGTTGGACAGTAGGGGAAG	*PARP14*	TCATATCTCTCTGGCTGCTCC	TCTTGGTCCAATGCAGTCCT	GGGTCAAAGAGATGGCAGGA	RP11-90P23
MetaEnhancer_2972_-	TGGTTGGACAGTAGGGGAAG	*PARP9*	ATCTCTTGGCCATGGAGCTT	TGAGGACCCTACTGTTGCTG	TTACAGGCACGTACCACCAT	RP11-90P23
MetaEnhancer_3039_+	AGCATTTAGGAGTGCACGTT	*SAMD9*	GATGTTAGGGGCTCTGCAGA	TGAGCACTTTGGAAGGCAAG	TTTGCTGTAACTGCCCTCCT	RP11-962H11
MetaEnhancer_502_+	CTGTCCCACACACCCCATAT	*TLR7-1*	CAGGAAGAGGGAGAGCAGAG	GAGGGTTTCATTTGCTGGGG	GTAAACCACTGCAGACTGGC	RP11-166I19
MetaEnhancer_341_-	CTGTCCCACACACCCCATAT	*TLR7-2*	CAGGAAGAGGGAGAGCAGAG	GAGGGTTTCATTTGCTGGGG	CATGCCAAGATCTGTAGACATCT	RP11-166I19
MetaEnhancer_116_+	TCTCCATGGGTCAGGTTGTT	*TNFSF10-1*	TACAGGTTCTTTGGTGCCCA	GAGCTGAGATCATGCACTGC	GCCATGCGCGGGATATAATC	RP11-240B20
MetaEnhancer_734_-	CTCCATGTTTCTCCCGTGTG	*TNFSF10-2*	TACAGGTTCTTTGGTGCCCA	GAGCTGAGATCATGCACTGC	GAGGACTACAGTAACGACCCT	RP11-183A2
MetaEnhancer_2005_+	GCAATAAACGTGGGAATGCC	*TNFSF10-3*	GAGCAGGACACGTAGACTCA	TGCCCATTTTCAGCATACAAAA	ACATTGTGCCCAGATGTTCC	RP11-259F22
MetaEnhancer_512_-	CACAGAAAGCATTGCCCCTT	*WDFY1*	AGGCTGGTCTTGAACTCCAA	TGTAGGAGGAGCAGGTTTGG	CTGGCAGACACCTCCACTTA	RP11-79C2

B. Control EP interaction

eRNA		Target genes			Control	BAC clone
Enhancer ID	eRNA - primer	Target gene name	Target promoter - primer1	Target promoter - primer2	Control primer	
MetaEnhancer_600_+	CTGGTCTTCTCTTCCCCACT	*APOBEC3B*	GCCTCAGCCTCTAGAGTAGC	CCTAGGGTAGCCTCACGTG	GGGATTACAGGTGCCCAGAA	RP11-358G23
MetaEnhancer_600_+	CTGGTCTTCTCTTCCCCACT	*APOBEC3D-1*	ACTCCCAACCTCATGATCCG	CACCTCTCTGTGCCTCTGAC	GGGATTACAGGTGCCCAGAA	RP11-358G23
MetaEnhancer_94_-	CTGCTGGTCTTCTCTTCCCC	*APOBEC3D-2*	TCGAACTCCCAACCTCATGA	CACCTCTCTGTGCCTCTGAC	ACAGAGTTCAGGACAGTGGT	RP11-358G23
MetaEnhancer_1529_+	ACAATCAGTCACTAGGAGGAGG	*CD2AP*	AAGGATTGGGGAGTCTCTCG	GTATGTGTGGGCATTTGTGC	TGGTAGGAGCCCAGATTCTG	RP11-947C14
MetaEnhancer_699_+	CCATGTTTGCTAGGCTGGTC	*DMXL1-1*	ATCCCTGCGGCCGAAATAT	CTGCATGCCAAACTTAAAACCT	CCCTGCTGCTTCCCTTTGAA	RP11-119J15
MetaEnhancer_454_-	CCATGTTTGCTAGGCTGGTC	*DMXL1-2*	TCCACACACTTCCTCTGGAC	AGAAAGGTGTTTGTGGTGCA	ATCCACAGAACCATGCTCCA	RP11-119J15
MetaEnhancer_475_+	CACTTCTCATTTCTCCAACCACA	*HLA-DMA*	GGCCGAAGTACCTAGCATGT	CAAGCTACTCAGGAGGCTGA	GACCCAGGAAGAGCTGATGT	RP11-260B12
MetaEnhancer_157_-	TCCAAACATGCAGCAGTCAC	*HLA-DRA*	TCCAAAGGCACCTGAATGAG	TCTGCTCAGGATCACTAGGT	CTGAAGAGTGACACCTCCTCA	RP11-379F19
MetaEnhancer_157_-	TCACCACCTTCCGGACTTTT	*HLA-DRB5*	ATTCCCCATACAGCACTTCC	TTGCTTCTCTGTTTTCTTTCCC	CTGAAGAGTGACACCTCCTCA	RP11-379F19
MetaEnhancer_820_-	CCAAAAGCACAAGACAGCCT	*IFI35*	GAGAGAGACCACAGCCCTTT	AAACTCTCCCACGTTCACCC	CCAGCCCCTTATGCCTCTTA	RP11-948G15
MetaEnhancer_311_+	CTGTGGGTCAAATGGGAGGA	*MLLT6*	TATTCCAGGGCCTAGAACGG	CATCTCCCTCTTGGCTTCCA	CAGCTTGAAGTGCCTTGTGG	RP11-607B2
MetaEnhancer_544_-	AGGGACAAGCAAGCATCTCT	*MYCBP2*	GAAGGGGTGGAGGTGAGTAC	CTCCTTCAGCCACTTCAGGA	TTTCATGGCTCCAACAACCT	RP11-775N1
MetaEnhancer_327_+	AACTTGAGCCCAGACAACCT	*PELI1*	CGATGCGTTTTCTTTATAGCCA	GCCCGGCCAAATATCAGTAG	CACTTCTGGTTTCTCATTCTCGA	RP11-46K17
MetaEnhancer_1477_-	GCTTGCCTCTCTTTGCCTTA	*SOCS1-1*	GCGGTCTTATGTGGTATGCC	AGTCAAGATCCTGGTGGCTT	CTCTGCCCAGCCTAGGAAC	RP11-697G17
MetaEnhancer_810_-	CCTTGGTTTCCTGGCCTCTA	*SOCS1-2*	AGTCAAGATCCTGGTGGCTT	GCGGTCTTATGTGGTATGCC	CAGGAGGCTCTGGGAAGAAT	RP11-697G17
MetaEnhancer_1228_+	TGCATGAGGCAGACTTGTTC	*ZBP1*	CCCAAGTCTCCCTTCTACCA	GTGTGAAGTCAGGTGCATGG	AACGGGAGCTTCGACTGTAA	RP11-1105M4 +RP11-877E5
MetaEnhancer_804_+	TGTGGTCCATATCCCGTAGA	*ZCCHC2*	CCAGGCTGGCAAATTGAGTT	CCACTCTTCAGCCTACTCGT	CAAGTCCTGCCCTGGTTTAC	RP11-645G19
MetaEnhancer_246_+	CACGCCCAGCTAATGTTTGT	*ZFAND6*	GCGGAGACTTTAAGGGCTTG	TTAAACATTTTAGCATCCCCAGG	GAACAAAAGCTCCTGAGGCC	RP11-916P11

Table S5. **siRNAs targeting eRNA of induced EP pairs.** siRNA name column contains assigned name for each siRNA pool. Target gene column contains gene names. Enhancer_ID column contains assigned Enhancer_ID for each eRNA tested. eRNA region column contains sequence of the high enrichment eRNA region from Table S2. siRNA ID contain unique identifier for each siRNAs tested. siRNA target sequence column contains DNA sequence of eRNA targeted by siRNAs. Sense column contains RNA sequence of the sense strand of siRNA duplex (5' to 3'). Antisense contains RNA sequence of the anti-sense strand of siRNA duplex (5' to 3').

siRNAs targeting eRNA of induced EP pairs

siRNA name	Target gene	Enhancer_ID	eRNA region	siRNA ID	siRNA target sequence	Sense	Antisense
siRNA#5A	*TNFSF10-1*	*MetaEnhancer_116_+*	TAAACCAGGATCTTCTTCAGCCTCCAAGGTAAGGAAATGTGTGATCTCCAAGCTCCCTCTTGTA<br>TGTATTGTGAACGCCACTGTCAGAAGAGAAACACCAAAGTTATTCACCTGGAAATGTTGCAGT<br>ATGAAGACCATGTATTTGATGGAGAGG	116A	GCCACTGTCAGAAGAGAAA	GCCACUGUCAGAAGAGAAA	UUUCUCUUCUGACAGUGGC
siRNA#5B				116B	TCTTCAGCCTCCAAGGTAA	UCUUCAGCCUCCAAGGUAA	UUACCUUGGAGGCUGAAGA
siRNA#5C				116C	GGAAATGTGTGATCTCCAA	GGAAAUGUGUGAUCUCCAA	UUGGAGAUCACACAUUUCC
siRNA#7A	*IRF8*	*MetaEnhancer_259_+*	GGCTCAGGCTGAGAGGATATTCTGCCGTTGTAGTTTTGCTCGGGGCCATTCGTTTTAAGAAGA<br>CTGGAGAGTCAGTTCCAGTTTGTCTTGGGGGACTAAGTTCTTATCATGTGGTTTCTACTGGTG<br>GCTTATTAGAACACATGCAGGTACAG	259A	GAGAGGATATTCTGCCGTT	GAGAGGAUAUUCUGCCGUU	AACGGCAGAAUAUCCUCUC
siRNA#7B				259B	GGTTTCTACTGGTGGCTTA	GGUUUCUACUGGUGGCUUA	UAAGCCACCAGUAGAAACC
siRNA#8A	*MLLT6*	*MetaEnhancer_311_+*	CCCAGCCCTCAGTGGCCCCACAGCAGCTTGGCTGTTCTTGGTTTTGTTTCTCTCTGCTTCTGCA<br>TGATATCTTTGAACAAAAAGTCCCAAGTGTACAAAAAGTCCCGAAAGGCGTTCGCAAACCACT<br>GACCTAGATGGAGGGAATTGTGAGGAGCAGAGGGCACCCTCTTATAAAATGCCTGTACTTCG<br>GTGCAGGGTTTGGTGGTGTCGGCGGTTTGGAGGCCCTTTAAGCTTCCTAACTCCTTGTCACTG<br>GTGGATGGTGGGGTGCCGGCAGGAGGGCATCCCTTTACATAGGGGCTCATTG	311A	CTCTGCTTCTGCATGATAT	CUCUGCUUCUGCAUGAUAU	AUAUCAUGCAGAAGCAGAG
siRNA#8B				311B	GAGGGCATCCCTTTACATA	GAGGGCAUCCCUUUACAUA	UAUGUAAAGGGAUGCCCUC
siRNA#10	*TLR7-1*	*MetaEnhancer_502_+*	GGGGAATGAGAAACAAAAGACAAGGTTAATTATGACACCGGGGCTTTACAATGCTAAAAATA<br>TCCTATATACAAAGGGATATGTAGGCTGTGTTCTTTTTCCATGTCATTACAAAGAACAGGCTCA<br>AGGTATCTGCAAATTTCTAATAAAAATATTATTACTTGAAAAATG	502A	GGCTCAAGGTATCTGCAAA	GGCUCAAGGUAUCUGCAAA	UUUGCAGAUACCUUGAGCC
siRNA#17A	*CD38*	*MetaEnhancer_1694_+*	GTCCACTTTTAGGAGTGTATGTACTTGGACACCTAAAAAATATGCTGCCACAAGAACATTTGTT<br>GTAGCATTctgTGCTCATTTATACAGGTCTAGTTAAGTAAACTCTAGCATACTA	1694A	AGCATTCTGTGCTCATTTA	AGCAUUCUGUGCUCAUUUA	UAAAUGAGCACAGAAUGCU
siRNA#17B				1694B	GAGTGTATGTACTTGGACA	GAGUGUAUGUACUUGGACA	UGUCCAAGUACAUACACUC
siRNA#23A	*CCR1*	*MetaEnhancer_258_-*	GCCTTTGAAAGTCTCGCATCTGCTGTTTTTCAGGTCTCCAAGTCCATTCTTTGTGTTTGGACTG<br>GTGAGTGTTTCTCACACTCTATAATCGCAAAGTAGGGAGGTATCTCTTCAAGAAGACAAGTGT<br>CATTCAAATATTTCTGCATAACAAACCAGACAAAACTTA	258A	GAGTGTTTCTCACACTCTA	GAGUGUUUCUCACACUCUA	UAGAGUGUGAGAAACACUC
siRNA#23B				258B	GGAGGTATCTCTTCAAGAA	GGAGGUAUCUCUUCAAGAA	UUCUUGAAGAGAUACCUCC
siRNA#24A	*TLR7-2*	*MetaEnhancer_341_-*	AGACTATTTATGCATGCATTGGTCTTTGAGCTGTTCCGCCTGCTGTCTTGTAAACTCAGAAGCA<br>GTTTTCCTGAGGTGGGTATTACTGACCATCTAGCTCACCATCTGAGACTCATAAGTAGATTTGT<br>GAGTGGTGAGGTGTAGTGAAGACCACTTGTTTGGGATTCAACATACATGCCAGCCAATGAAA<br>ATGTTAACTCACATCTGCATATGCCTTCATCTTGTTATGGAACTGGAGGCACCATCATTCATAAT<br>GCTTAAAAATAAAACACCTGCA	341A	GAGGCACCATCATTCATAA	GAGGCACCAUCAUUCAUAA	UUAUGAAUGAUGGUGCCUC
siRNA#24B				341B	CTCACCATCTGAGACTCAT	CUCACCAUCUGAGACUCAU	AUGAGUCUCAGAUGGUGAG
siRNA#28A	*WDFY1*	*MetaEnhancer_512_-*	GTTAGGTTACATATCAAGTTAGGTTCACTGTGTACTGAAAAACCTTTAGGCCAAAATTAAAATA<br>TGTACAACGGAGGCTTTAGTCCAAACTTAATTTAACAGTACTACAATTAAGTATCAGTATTGCC<br>ATAATGTATCAGTCAGGGT	512A	GGCTTTAGTCCAAACTTAA	GGCUUUAGUCCAAACUUAA	UUAAGUUUGGACUAAAGCC
siRNA#28B				512B	GCCATAATTGTATCAGTCA	GCCAUAAUUGUAUCAGUCA	UGACUGAUACAAUUAUGGC
siRNA#28C				512C	TAGGTTCACTGTGTACTGA	UAGGUUCACUGUGUACUGA	UCAGUACACAGUGAACCUA
siRNA#30	*TNFSF10-2*	*MetaEnhancer_734_-*	GCTTGGACCCACAGTACAGATCACCCATCTTGGGCCTATCACACTGAGCAATTGCTGACCTGC<br>ATCTCTGTGGAGTGGAGCCCCCAGGAGACAAGTAAAAGACCCCCAGCCACAAC	734A	CACACTGAGCAATTGCTGA	CACACUGAGCAAUUGCUGA	UCAGCAAUUGCUCAGUGUG
siRNA#34A	*IFNb1*	*MetaEnhancer_1571_+*	TTCTTCTGGCTGCCTGGAAGATGCCTCTGTTATTTTAGTGAAAACATTTAGCTCCTCGAAAGGT<br>AGAGAGAACCAAGAGGTAAAGTGTGCCATTGTAGACAGCTGGTGTGTGTGCCAGGTAGCAG<br>TGCTTCTGTCTACATCCTGGGATATT	1571A	CTGTCTACATCCTGGGATA	CUGUCUACAUCCUGGGAUA	UAUCCCAGGAUGUAGACAG
siRNA#34B				1571B	TAAAGTGTGCCATTGTAGA	UAAAGUGUGCCAUUGUAGA	UCUACAAUGGCACACUUUA
siRNA#35A	*IFI16*	*MetaEnhancer_1136_+*	TTTCTGCTATCTAGATCTTTGACTACCAACCAGCCAGGAGTGGTCTGCCTCTTCTTTGGTCTGA<br>GCTTGCCTTGTGCTTATATTGTTGTACCCAAGCGAGT	1136A	CTTATATTGTTGTACCCAA	CUUAUAUUGUUGUACCCAA	UUGGGUACAACAAUAUAAG
siRNA#35B				1136B	CTATCTAGATCTTTGACTA	CUAUCUAGAUCUUUGACUA	UAGUCAAAGAUCUAGAUAG
siRNA#36A	*PARP14*	*MetaEnhancer_2972_-*	CGCGACCGTTCCATTCTTGAGTTTCTTCACTTTGCCAGCTTGGGTGTCTCTCCCGAGGCCGTC<br>GTTCTAGTTATGGCTCATCCAGCTGACGCTGCAACTTCTCTTGTTCTGTAGCATTTCTTCTACTT<br>CTCTTTCTCTACCAGCTGTGTCAACCGGCT	2972A	CAACTTCTCTTGTTCTGTA	CAACUUCUCUUGUUCUGUA	UACAGAACAAGAGAAGUUG
siRNA#36B				2972B	CTCTTGTTCTGTAGCATTT	CUCUUGUUCUGUAGCAUUU	AAAUGCUACAGAACAAGAG
siRNA#36C				2972C	CCGTTCCATTCTTGAGTTT	CCGUUCCAUUCUUGAGUUU	AAACUCAAGAAUGGAACGG
siRNA#37A	*CD38*	*MetaEnhancer_3795_-*	CACATCTCACCCCCAGACCTGCCATTGGCATGCACATCTTTAACCACAAACCACGATTGAAAAT<br>GAGAAAGAAAACTCTCCTCTAGCTGGGCGCTTCATAGGACTAGCCAGGTACGACCATCTCTG<br>AGGGTCTTTGACAGCTCAGTTTTACTGCATAATCTGTTTTCAAGACCCTTTAAAAGACCAGAT	3795A	GAGGGTCTTTGACAGCTCA	GAGGGUCUUUGACAGCUCA	UGAGCUGUCAAAGACCCUC
siRNA#37B				3795B	CTGCCATTGGCATGCACAT	CUGCCAUUGGCAUGCACAU	AUGUGCAUGCCAAUGGCAG
siRNA#37C				3795C	GCATGCACATCTTTAACCA	GCAUGCACAUCUUUAACCA	UGGUUAAAGAUGUGCAUGC
siRNA#38A	*TNFSF10-3*	*MetaEnhancer_2005_+*	CATAACTACCAGAAGCCATTGGTGGCAAGATCTCAATCAGGACATGTCGGTACAATGCTCTGA<br>TTATCTCGTCTGCATCCCTGATTACTTATGGTCCAGATATAAACTACT	2005A	CTGATTATCTCGTCTGCAT	CUGAUUAUCUCGUCUGCAU	AUGCAGACGAGAUAAUCAG
siRNA#38B				2005B	CTGCATCCCTGATTACTTA	CUGCAUCCCUGAUUACUUA	UAAGUAAUCAGGGAUGCAG
siRNA#38C				2005C	TACTTATGGTCCAGATATA	UACUUAUGGUCCAGAUAUA	UAUAUCUGGACCAUAAGUA
siRNA#41A	*SAMD9*	*MetaEnhancer_3039_+*	GTCCTTAAGGCTGATGTGAATCTCTGCCCTTTCCAATGTGTCATGTGATTGCTCCAAATTAAAA<br>CACCTATTATTATTATTTTG	3039A	GAATCTCTGCCCTTTCCAA	GAAUCUCUGCCCUUUCCAA	UUGGAAAGGGCAGAGAUUC
siRNA#41B				3039B	TCCAATGTGTCATGTGATT	UCCAAUGUGUCAUGUGAUU	AAUCACAUGACACAUUGGA
siRNA#42A	*KIAA0040*	*MetaEnhancer_1115_+*	CACCTCACTTCTGTTTTCTGTCCATTAATGCTTCCTGCCCACGTTGTGGGGAGGAGCTCTCTGA<br>ACCTCTGCTGCTTCTGAGGGATTCAAGAATATATTTTTTGCTCAATTAAACTCCTAAATCTAATTT<br>GTCTAAAGCTTTTCTT	1115A	CAATTAAACTCCTAAATCT	CAAUUAAACUCCUAAAUCU	AGAUUUAGGAGUUUAAUUG
siRNA#42B				1115B	CTGAGGGATTCAAGAATAT	CUGAGGGAUUCAAGAAUAU	AUAUUCUUGAAUCCCUCAG
siRNA#42C				1115C	CTGAACCTCTGCTGCTTCT	CUGAACCUCUGCUGCUUCU	AGAAGCAGCAGAGGUUCAG
siRNA#43A	*PARP9*	*etaEnhancer_2972_-*	ACTTCCTGTCCGCGACCGTTCCATTCTTGAGTTTCTTCACTTTGCCAGCTTGGGTGTCTCTCCC<br>GAGGCCGTCGTTCTAGTTATGGCTCATCCAGCTGACGCTGCAACTTCTCTTGTTCTGTAGCATT<br>TCTTCTACTTCTCTTTCTCTACCAGCTGTGTCAACCGGCTTTG	2972A	CAACTTCTCTTGTTCTGTA	CAACUUCUCUUGUUCUGUA	UACAGAACAAGAGAAGUUG
siRNA#43B				2972B	CCGTTCCATTCTTGAGTTT	CCGUUCCAUUCUUGAGUUU	AAACUCAAGAAUGGAACGG
siRNA#46A	*ZBP1*	*MetaEnhancer_1228_+*	TGATTACCTCACTCTCATGCAGGGCTGAATATTACTGTTTCCCCTGTTAAACAAGGGTGTATTA<br>CTTCCGAAATCTGACAACCCCAAGCACCAAAAGGTTTAAAAATATCTCGAGATTGTAAAGCCT<br>CCGACAGAATGCTGAAACAGGATTGCACAGTTGACCAGGAGCTTCTGAGGTTGTGGCGGAC<br>CCTCCATGTTTCTGACTGCCGGACAGTCACAGCCCCCTCTTCCCTAATGCCACCAGATGTTCCC<br>TGGGACACCTGCC	1228A	CCGACAGAATGCTGAAACA	CCGACAGAAUGCUGAAACA	UGUUUCAGCAUUCUGUCGG
siRNA#46A				1228B	GGGTGTATTACTTCCGAAA	GGGUGUAUUACUUCCGAAA	UUUCGGAAGUAAUACACCC
siRNA#46A				1228C	CTCATGCAGGGCTGAATAT	CUCAUGCAGGGCUGAAUAU	AUAUUCAGCCCUGCAUGAG
siRNA#47&48A	*MNDA & ACKR1*	*MetaEnhancer_1136_+*	TTTCTGCTATCTAGATCTTTGACTACCAACCAGCCAGGAGTGGTCTGCCTCTTCTTTGGTCTGA<br>GCTTGCCTTGTGCTTATATTGTTGTACCCAAGCGAGT	1136A	CTTATATTGTTGTACCCAA	CUUAUAUUGUUGUACCCAA	UUGGGUACAACAAUAUAAG
siRNA#47&48B				1136B	CTATCTAGATCTTTGACTA	CUAUCUAGAUCUUUGACUA	UAGUCAAAGAUCUAGAUAG

Table S6. siRNAs targeting eRNA of control EP pairs. siRNA name column contains assigned name for each siRNA pool. Target gene column contains gene names. Enhancer_ID column contains assigned Enhancer_ID for each eRNA tested. eRNA region column contains sequence of the high enrichment eRNA region for each eRNA. siRNA ID contain unique identifier for each siRNAs tested. siRNA target sequence column contains DNA sequence of eRNA targeted by siRNAs. Sense column contains RNA sequence of the sense strand of siRNA duplex (5' to 3'). Antisense contains RNA sequence of the anti-sense strand of siRNA duplex (5' to 3').

siRNAs targeting of control EP pairs

siRNA name	Target gene	Enhancer_ID	eRNA region	siRNA ID	siRNA target sequence	Sense	Antisense
siRNA#1A	*HLA-DMA*	*MetaEnhancer_475_+*	ACAGTCTACAAAGAGGCCGTGGAAGCTGTCGGGGAAGGAGAATGTTCAAGTAGCACAGGCAATCAAACACTTCCTATTGCTCCAGGTGCCAAAGCAGGAATGAAAACCTGTCCCCTCTGTTGAATACTCTTCTTCTTCACTCCTAAAACTACACACCTGATGTTAGTCGTCAGCCCTCTTCTTATCACTCTACACCTGCTGCTCTGGAGAACTCATCCAGGCCTGTGGCTCCCTGCACGTCTACACTAGTAACCTCTGAATCCACGGTCTCCAGCACTCCCTCCTGCTCCCATCCCCAGGTGGCAGTCAGGT	475A	CACCTGATGTTAGTCGTCA	CACCUGAUGUUAGUCGUCA	UGACGACUAACAUCAGGUG
siRNA#1B				475B	CTCTTCTTCTTCACTCCTA	CUCUUCUUCUUCACUCCUA	UAGGAGUGAAGAAGAAGAG
siRNA#2&3A	*HLA-DRA & DRB5*	*MetaEnhancer_157_-*	GAATTCAAAGGGTCTCTTCTAGAGGATCCTGGGTTATGTCCTCCACAGGAACTTTGGTGTTGGCCCCTCTTCCTCAAATGTGAGGATGTACCAATGGCCTCCCCATTATCTCCTTTCTTTTTCTTTCTAACTCCAATGTTTATAAAGCCTATATCCCTGTAGTGTATGTAGGTTCTCTGACAGAAGTTATACTTAGTGCTCTGTCTTTCTTATGGGGAAAAATCCCTGGAACTGAAGCTAAGATCTTTAGTACTTGGAGTCACCCTACAGATA	157A	CTTTCTAACTCCAATGTTT	CUUUCUAACUCCAAUGUUU	AAACAUUGGAGUUAGAAAG
siRNA#2&3B				157B	GTATGTAGGTTCTCTGACA	GUAUGUAGGUUCUCUGACA	UGUCAGAGAACCUACAUAC
siRNA#2&3C				157C	CCTCAAATGTGAGGATGTA	CCUCAAAUGUGAGGAUGUA	UACAUCCUCACAUUUGAGG
siRNA#6A	*ZFAND6*	*MetaEnhancer_246_+*	ATGGTTTGTTTTCCCCTCTCCCTGCTAGAAGCACAAGGGGATTTTTCTCTGATAATCATTGTGAGAACCTGTTAGAACTCCTGGAGGTAAAACTCAAAAGTATGGCGGCCCCCCTGAATGAGTCTTCCTGGAATTAACTGTCAAACTTGCCACTCTGAGTCTCCAGCAATTCTTGAATTACAATTCAGATTTTCCTATCCTGGTACAGGTTCCTGT	246A	CTCTGAGTCTCCAGCAATT	CUCUGAGUCUCCAGCAAUU	AAUUGCUGGAGACUCAGAG
siRNA#6B				246B	GTGAGAACCTGTTAGAACT	GUGAGAACCUGUUAGAACU	AGUUCUAACAGGUUCUCAC
siRNA#9A	*PELI1*	*MetaEnhancer_327_+*	TACCAATGTCTCTGGGCCTTGCTCTACTAGTACAACAGAGGAGAGAGAAATTCTAGAAGATTTTCAACTCCCCTCCTGCTCTGTAACTCTGGTTCGCTATGTGCTAAATGCACCTTGAAATAAACACTTCCTTTGTGTGTGTGTGTTGCGCGGTGGAATCCTCACTTTACAGAAGAGGA	327A	CTGTAACTCTGGTTCGCTA	CUGUAACUCUGGUUCGCUA	UAGCGAACCAGAGUUACAG
siRNA#9B				327B	GAGAGAAATTCTAGAAGAT	GAGAGAAAUUCUAGAAGAU	AUCUUCUAGAAUUUCUCUC
siRNA#11&12A	*APOBEC3D-1 & APOBEC3B*	*MetaEnhancer_600_+*	CACTTGCTTCTAGGCTGAGGAGGGCGGGGCTGTTGTCAGAGCCCAGAATCAAAGCCAGAGGAGCAGGTGGACGCTGAGACTGTCCCCTCACCCTGCTCCACGGGCAATGTTGAAGTGGGCATCTGGGTGTT	600A	TTGTCAGAGCCCAGAATCA	UUGUCAGAGCCCAGAAUCA	UGAUUCUGGGCUCUGACAA
siRNA#11&12B				600B	GCTCCACGGGCAATGTTGA	GCUCCACGGGCAAUGUUGA	UCAACAUUGCCCGUGGAGC
siRNA#13A	*DMXL1-1*	*MetaEnhancer_699_+*	ACCATGACTCACCCTCTTTCCTCGTCACCTGCTAGTCCTCCCATTCACTCCCCTTCAGTCATATTGGCCTTGCTTCTCTTCAGACGGGCCAGCCACACTCAGGGCCTTTGCACTGACTATTCCTTCTGCCTGGAATGTTCCTCCTCCAAGTATCCATATGGCTAACTCCCTCAATTCTTTGAGATCTTTAACCACAAGGCGCTTTCCCAAATAAGTCTTCTCTGGCTACCCTTTTTAAAATTTTAACCCCCACCCTTCACATTTTATATACTCCCCTTCCTTGCCTTTTTTCAGCTTCTTGTC	699A	CTAACTCCCTCAATTCTTT	CUAACUCCCUCAAUUCUUU	AAAGAAUUGAGGGAGUUAG
siRNA#13B				699B	CTCGTCACCTGCTAGTCCT	CUCGUCACCUGCUAGUCCU	AGGACUAGCAGGUGACGAG
siRNA#14A	*ZCCHC2*	*MetaEnhancer_804_+*	CAATCGCTGCCTGAGTACCTATGTGTTCGTGGCTTGATTTACCTTATCTGTAATTCCTGGATGTTAAAAGGACACCAAAAATCTCTGACACCCTGATTAGCCTACCTTGAAGAACCCAAGCCCAGGATTTCTGCCTTTGCCTAGAAAAAGGACTGATTCAGAACTTCCTGAGCTACCTAGTATT	804A	GTGTTCGTGGCTTGATTTA	GUGUUCGUGGCUUGAUUUA	UAAAUCAAGCCACGAACAC
siRNA#14B				804B	TTCAGAACTTCCTGAGCTA	UUCAGAACUUCCUGAGCUA	UAGCUCAGGAAGUUCUGAA
siRNA#14C				804C	CTGACACCCTGATTAGCCT	CUGACACCCUGAUUAGCCU	AGGCUAAUCAGGGUGUCAG
siRNA#16A	*CD2AP*	*MetaEnhancer_1529_+*	CACAATGAGAGTTGTCCCTAGATTTCACTGGAATGTAAATAAAAGCCTTGATCATTCTCACTGCTCGACAGCCACAGTTTCACTTTGTTCTTCATGGCTCTGCCAACCCTCTGAAACTCTCAGTCACGGGGACTGTGCTCTCAGCTTTCCTCCAAGAATTTCTCCACTGCCATTGCCTCCTGTTTTTT	1529A	CAGCTTTCCTCCAAGAGAT	CAGCUUUCCUCCAAGAGAU	AUCUCUUGGAGGAAAGCUG
siRNA#16B				1529B	GCCACAGTTTCACTTTGTT	GCCACAGUUUCACUUUGUU	AACAAAGUGAAACUGUGGC
siRNA#16C				1529C	CTCTGCCAACCCTCTGAAA	CUCUGCCAACCCUCUGAAA	UUUCAGAGGGUUGGCAGAG
siRNA#18A	*APOBEC3D-2*	*MetaEnhancer_94_-*	ATCCTCTGACTTGGGGCAGAGCTGAGGTAGACATCTGGGTGTGTCTGGGAAACCCCGGGGAAGGTTCCTTTCTGTCCTGTCACTGTCTGTGTGTGCTTATGTGTCTGTGTGTGTCTGTGTCTTTGCCACCAGAAGGAGTTGGGCCTGTTTGCTCATGAGCAGCTGTCGAGGAGGCCCACTGTGTACCACATATGCGGCTCCTGAGG	94A	CACTGTCTGTGTGTGCTTA	CACUGUCUGUGUGUGCUUA	UAAGCACACACAGACAGUG
siRNA#18B				94B	CTGTGTCTTTGCCACCAGA	CUGUGUCUUUGCCACCAGA	UCUGGUGGCAAAGACACAG
siRNA#23A	*CCR1*	*MetaEnhancer_258_-*	GCCTTTGAAAGTCTCGCATCTGCTGTTTTTCAGGTCTCCAAGTCCATTCTTTGTGTTTGGACTGGTGAGTGTTTCTCACACTCTATAATCGCAAAGTAGGGAGGTATCTCTTCAAGAAGACAAGTGTCATTCAAATATTTCTGCATAACAAACCAGACAAAACTTA	258A	GAGTGTTTCTCACACTCTA	GAGUGUUUCUCACACUCUA	UAGAGUGUGAGAAACACUC
siRNA#23B				258B	GGAGGTATCTCTTCAAGAA	GGAGGUAUCUCUUCAAGAA	UUCUUGAAGAGAUACCUCC
siRNA#26A	*DMXL1-2*	*MetaEnhancer_454_-*	CTATCTCTCTCTTTCATGGGTCAGTTTCTATTTCCCTCTGTGTTTCTGTTCATTGTCTTCCTGGTGGCTAACTTCTGCTTATAGTTTCTGCTCCCTTTTAACTTCTGTGTGCACATGACTTGGACTTGTTATGGTACTATGCTCTGTGCTGGAAACGTAGCGGTGAGTAAGGCAGCCCTGACTTCTCAGCTCTGCATAGGACCCACAGTAGGGTAGGGAATAAACACACACACACACACACACACACACACAGTTGTGATGTGCTATGA	454A	CTCTCTCTTTCATGGGTCA	CUCUCUCUUUCAUGGGUCA	UGACCCAUGAAAGAGAGAG
siRNA#26B				454B	CTGTGTTTCTGTTCATTGT	CUGUGUUUCUGUUCAUUGU	ACAAUGAACAGAAACACAG
siRNA#26C				454C	CCCACAGTAGGGTAGGGAA	CCCACAGUAGGGUAGGGAA	UUCCCUACCCUACUGUGGG
siRNA#29A	*MYCBP2*	*MetaEnhancer_544_-*	AGGAACTAGCTAACATAGGTTTTGTTATCTGCTTCGAGTGTGCTCTGTGTGCATCCACACCCAAGTCCCAGGCCCCAAGAGTCTGTGTGGAGCTGTGTGATGGGCTGATGGTACCTCTTACATGAGGCCTTTTGGGAGATACTGAGAAAGCACTGACTAGGAGTTGAGGCTCCACCCCAGATTAGTCATCATCTGTGTGT	544A	GATGGTACCTCTTACATGA	GAUGGUACCUCUUACAUGA	UCAUGUAAGAGGUACCAUC
siRNA#29B				544B	TGCTCTGTGTGCATCCACA	UGCUCUGUGUGCAUCCACA	UGUGGAUGCACACAGAGCA
siRNA#29C				544C	GGGAGATACTGAGAAAGCA	GGGAGAUACUGAGAAAGCA	UGCUUUCUCAGUAUCUCCC
siRNA#33	*SOCS1-1*	*MetaEnhancer_1477_-*	TGTAATTATTTGTTTACTGTAGGCCTCCCCTTTGATGAGGAGCCCCCTGGGAGTGTCAGGCTCTCTGCTGTCCCCCCAGCACCAGCACAAGGC	1477A	GTGTCAGGCTCTCTGCTGT	GUGUCAGGCUCUCUGCUGU	ACAGCAGAGAGCCUGACAC
siRNA#39A	*SOCS1-2*	*MetaEnhancer_810_-*	TTAGTTTCAGTTCTTTGATACTTTTTTGAGAGGCCTGAAGGTCCTTTCCTGATATAGAACTCACGTAAACAAATAAAAGCTTCAAGTTTTAAGACAAGAAGGGTCAATTTCTTTGTTTATCCAAAAAACTATCTA	810A	GGGTCAATTTCTTTGTTTA	GGGUCAAUUUCUUUGUUUA	UAAACAAAGAAAUUGACCC
siRNA#39B				810B	GTCCTTTCCTGATATAGAA	GUCCUUUCCUGAUAUAGAA	UUCUAUAUCAGGAAAGGAC
siRNA#39C				810C	CAATTTCTTTGTTTATCCA	CAAUUUCUUUGUUUAUCCA	UGGAUAAACAAAGAAAUUG
siRNA#40A	*IFI35*	*MetaEnhancer_820_-*	CCTACACTGAAAGCCCATGGGGTTGAAGCAGGATTTGGTTCACGCTAGAACTTCTGAGAGTCAGTGTGTATCTTGATAAACAGCAACAGAGCTTAGTCATGAGTTCCCTGTAACTGGCTGCTCAGAGAGTTTGCTCCCCACCTCCTGGGGAGAACTTACCTGGGAAGAGGCCAATGTTTCTATCTAAGCCTGTCCCGTCCTCTGAGTTTCCAACCTTCTAATTTCACGTTGGGAGTGCCTC	820A	CAGTGTGTATCTTGATAAA	CAGUGUGUAUCUUGAUAAA	UUUAUCAAGAUACACACUG
siRNA#40B				820B	GAGTCAGTGTGTATCTTGA	GAGUCAGUGUGUAUCUUGA	UCAAGAUACACACUGACUC
siRNA#46A	*ZBP1*	*MetaEnhancer_1228_+*	TGATTACCTCACTCTCATGCAGGGCTGAATATTACTGTTTCCCCTGTTAAACAAGGGTGTATTACTTCCGAAATCTGACAACCCCAAGCACCAAAAGGTTTAAAAATATCTCGAGATTGTAAAGCCTCCGACAGAATGCTGAAACAGGATTGCACAGTTGACCAGGAGCTTCTGAGGTTGTGGCGGACCCTCCATGTTTCTGACTGCCGGACAGTCACAGCCCCCTCTTCCCTAATGCCACCAGATGTTCCCTGGGACACCTGCC	1228A	CCGACAGAATGCTGAAACA	CCGACAGAAUGCUGAAACA	UGUUUCAGCAUUCUGUCGG
siRNA#46A				1228B	GGGTGTATTACTTCCGAAA	GGGUGUAUUACUUCCGAAA	UUUCGGAAGUAAUACACCC
siRNA#46A				1228C	CTCATGCAGGGCTGAATAT	CUCAUGCAGGGCUGAAUAU	AUAUUCAGCCCUGCAUGAG

Table S7. **eRNA and target gene expression fold changes after eRNA KD with siRNA**. siRNA name column contains assigned name for each siRNA used. Distance column contains approximate distance between enhancer and target gene promoter. fold changes of eRNA expression fold column contains normalized fold change in eRNA expression upon eRNA KD. mRNA expression fold column contains normalized fold change in mRNA expression of the target gene upon eRNA KD.

.

eRNA expression and mRNA expression fold change after eRNA KD with siRNA

siRNA name	Target gene	Enhancer_ID	Distance	eRNA expression fold	mRNA expression fold
#1A siRNA	*HLA-DMA*	*MetaEnhancer_475_+*	75000	0	1.423721208
#1B siRNA			75000	0.540862333	0.878430468
#2A siRNA	*HLA-DRA*	*MetaEnhancer_157_-*	26000	2.163449332	1.016281451
#2B siRNA			26000	0.615600653	0.668948321
#2C siRNA			26000	1.689660424	0.366012967
#3A siRNA	*HLA-DRB5*	*MetaEnhancer_157_-*	44000	2.163449332	0.79922115
#3B siRNA			44000	0.615600653	0.628506687
#3C siRNA			44000	1.689660424	0.268563162
#5AsiRNA	*TNFSF10-1*	*MetaEnhancer_116_+*	69000	0.60149862	0.695730272
#5BsiRNA			69000	0.684600064	0.874542257
#5CsiRNA			69000	0.77916458	1.228246391
#6A siRNA	*ZFAND6*	*MetaEnhancer_246_+*	34000	2.518677954	0.963707118
#6B siRNA			34000	0.827405623	0.33140486
#7AsiRNA	*IRF8*	*MetaEnhancer_259_+*	73000	0.707106781	0.649169294
#7BsiRNA			73000	1.194687532	0.52123288
#8AsiRNA	*MLLT6*	*MetaEnhancer_311_+*	50000	2.795810671	0.831237896
#8BsiRNA			50000	7.193364285	2.434007027
#9A siRNA	*PELI1*	*MetaEnhancer_327_+*	82000	1.713096791	2.750969224
#9B siRNA			82000	0.983915733	1.018613446
#10 siRNA	*TLR7-1*	*MetaEnhancer_502_+*	20000	0.631417726	2.934944726
#11A siRNA	*APOBEC3D-1*	*MetaEnhancer_600_+*	65000	1.580082624	1.701334322
#11B siRNA			65000	0.763129604	1.990779358
#12A siRNA	*APOBEC3B*	*MetaEnhancer_600_+*	26000	1.580082624	1.2397077
#12B siRNA			26000	0.381564802	0.552227
#13A siRNA	*DMXL1-1*	*MetaEnhancer_699_+*	37000	0.625320044	1.815038311
#13B siRNA			37000	1.006722921	1.624504793
#14AsiRNA	*ZCCHC2*	*MetaEnhancer_804_+*	15000	1.447336117	5.277909696
#14BsiRNA			15000	6.573826285	6.276527763
#14CsiRNA			15000	0	4.469045294
#16A siRNA	*CD2AP*	*MetaEnhancer_1529_+*	62000	2.417082417	1.781797436
#16B siRNA			62000	0.990777721	1.74916534
#16C siRNA			62000	0.692538733	1.280463977
#17A siRNA	*CD38-1*	*MetaEnhancer_1694_+*	5500	0.409896999	1.040059934
#17B siRNA			5500	3.863745316	0.369249191
#18A siRNA	*APOBEC3D-2*	*MetaEnhancer_94_-*	65000	0.287114879	1.594790224
#18B siRNA			65000	2.356588726	3.256600313
#23A siRNA	*CCR1*	*MetaEnhancer_258_-*	91000	2.328929014	0.604270604
#23B siRNA			91000	3.49833068	0.759576231
#24A siRNA	*TLR7-2*	*MetaEnhancer_341_-*	22000	6.320330495	8.186991136
#24B siRNA			22000	0.761368436	1.487957514
#26A siRNA	*DMXL1-2*	*MetaEnhancer_454_-*	38000	0.835087919	1.353473524
#26B siRNA			38000	3.792283097	1.543993487
#26C siRNA			38000	0	4.9588308
#28A siRNA	*WDFY1*	*MetaEnhancer_512_-*	2600	0.174742204	0.104627452
#28B siRNA			2600	1.522736872	2.075319318
#28C siRNA			2600	0.540862333	0.169184191
#29A siRNA	*MYCBP2*	*MetaEnhancer_544_-*	87000	3.023039857	0.664327558
#29B siRNA			87000	4.845458993	1.11984573
#29C siRNA			87000	0.422395587	0.084785811
#30 siRNA	*TNFSF10-2*	*MetaEnhancer_734_-*	67000	0.835087919	0.733736182
#34A siRNA	*IFNb1*	*MetaEnhancer_1571_+*	18000	0	0.502315837
#34B siRNA			18000	0.410845157	0.57567774
#35A siRNA	*IFI16*	*MetaEnhancer_1136_+*	2300	0.23648036	0.439316726
#35B siRNA			2300	0.34268249	1.25411241
#36A siRNA	*PARP14*	*MetaEnhancer_2972_-*	18000	0.434280777	0.993115441
#36B siRNA			18000	0.720315074	1.887792242
#36C siRNA			18000	0.699001117	0.667435348
#37A siRNA	*CD38-2*	*MetaEnhancer_3795_-*	22000	1.032923445	1.135504429
#37B siRNA			22000	0.779182582	0.562529242
#37C siRNA			22000	0.240376317	0.92444966
#38AsiRNA	*TNFSF10-3*	*MetaEnhancer_2005_+*	27000	0.801069878	0.279954964
#38BsiRNA			27000	0.92873141	1.219762273
#38CsiRNA			27000	0.930879716	0.391368623
#39A siRNA	*SOCS1-2*	*MetaEnhancer_810_-*	48400	1.587364376	2.244872228
#39B siRNA			48400	1.498272459	3.160092233
#39C siRNA			48400	1.674000548	1.286364948
#40A siRNA	*IFI35*	*MetaEnhancer_820_-*	77000	0.550952558	0.237061508
#40B siRNA			77000	0.217637641	0.390483457
#41A siRNA	*SAMD9*	*MetaEnhancer_3039_+*	62000	0.848664754	0.304243849
#41B siRNA			62000	0.168007786	0.281908718
#42A siRNA	*KIAA0040*	*MetaEnhancer_1115_+*	94000	0.187712939	0.554425941
#42B siRNA			94000	0.295917448	0.255678012
#42C siRNA			94000	0.251146313	0.425058505
#43A siRNA	*PARP9*	*etaEnhancer_2972_-*	96000	0.297982601	0.299369676
#43B siRNA			96000	0.751059963	0.402390086
#46A siRNA	*ZBP1*	*MetaEnhancer_1228_+*	120000	0.227930622	0.903335201
#46B siRNA			120000	5.051341805	2.06575143
#46C siRNA			120000	0.082469244	0.342300032
#47A siRNA	*MNDA*	*MetaEnhancer_1136_+*	156000	0.23981603	0.430275719
#47B siRNA			156000	0.273573425	0.733736182
#48A siRNA	*ACKR1*	*MetaEnhancer_1136_+*	196000	0.23981603	0.673616788
#48B siRNA			196000	0.273573425	0.842841545

Table S8. eRNA primers. Target gene column contains gene names. Enhancer_ID column contains assigned Enhancer_ID for each eRNA tested. eRNA-F column contains name of forward primers used for realtime PCR detection of eRNA expression. Forward primer sequence column contains forward primer seuqences. eRNA-R column contains name of reverse primers used for realtime PCR detection of eRNA expression. Forward primer sequence column contains reverse primer seuqences.

eRNA primers

Target gene	Enhancer_ID	eRNA-F	Forward primer sequence	eRNA-R	Reverse primer sequence
HLA-DMA	*MetaEnhancer_475_+*	eRNA#1A-F	CTTCTTCACTCCTAAAACTACACACC	eRNA#1A-R	AGAGTGATAAGAAGAGGGCTGA
		eRNA#1B-F	AATGAAAACCTGTCCCCTCTG	eRNA#1B-R	AGGTGTGTAGTTTTAGGAGTGAAGAA
HLA-DRA & DRB5	*MetaEnhancer_157_-*	eRNA#2-3A-F	CTCCCCATTATCTCCTTTCTTTT	eRNA#2-3A-R	AGGGATATAGGCTTTATAAACATTGG
		eRNA#2-3B-F	GTGTATGTAGGTTCTCTGACAGAAGT	eRNA#2-3B-R	TTTTTCCCCATAAGAAAGACAGA
		eRNA#2-3C-F	TCCTCCACAGGAACTTTGGT	eRNA#2-3C-R	GGCCATTGGTACATCCTCAC
TNFSF10-1	*MetaEnhancer_116_+*	eRNA#5A-F	TTGTATGTATTGTGAACGCCACT	eRNA#5A-R	CATTTCCAGGTGAATAACTTTGG
		eRNA#5BC-F	AGGATCTTCTTCAGCCTCCA	eRNA#5BC-R	AATACATACAAGAGGGAGCTTGG
ZFAND6	*MetaEnhancer_246_+*	eRNA#6A-F	TCTTCCTGGAATTAACTGTCAAA	eRNA#6A-R	TGTAATTCAAGAATTGCTGGAGAC
		eRNA#6B-F	GGGATTTTTCTCTGATAATCATTGTG	eRNA#6B-R	TTGAGTTTTACCTCCAGGAGTTCT
IRF8	*MetaEnhancer_259_+*	eRNA#7A-F	TGAGAGGATATTCTGCCGTTG	eRNA#7A-R	GAACTGACTCTCCAGTCTTCTTAAA
		eRNA#7B-F	TGGGGGACTAAGTTCTTATCATGT	eRNA#7B-R	CCTGCATGTGTTCTCAATAAGC
MLLT6	*MetaEnhancer_311_+*	eRNA#8A-F	TGGCTGTTCTTGGTTTTGTTT	eRNA#8A-R	CGGGACTTTTTGTACACTTGG
		eRNA#8B-F	ACTCCTTGTCACTGGTGGATG	eRNA#8B-R	GCCCCTATGTAAAGGGATGC
PELI1	*MetaEnhancer_327_+*	eRNA#9A-F	TTTTCAACTCCCCTCCTGCT	eRNA#9A-R	TTTCAAGGTGCATTTAGCACA
		eRNA#9B-F	TCTCTGGGCCTTGCTCTACT	eRNA#9B-R	GCAGGAGGGGAGTTGAAAAT
TLR7-1	*MetaEnhancer_502_+*	eRNA#10-F	AGGCTGTGTTCTTTTTCCATGT	eRNA#10-R	AGAAATTTGCAGATACCTTGAGC
APOBEC3D-1 & APOBEC3B	*MetaEnhancer_600_+*	eRNA#11-12A-F	CACTTGCTTCTAGGCTGAGGA	eRNA#11-12A-R	TCCTCTGGCTTTGATTCTGG
		eRNA#11-12B-F	AGGTGGACGCTGAGACTGT	eRNA#11-12B-R	CCAGATGCCCACTTCAACAT
DMXL1-1	*MetaEnhancer_699_+*	eRNA#13A-F	CTGGAATGTTCCTCCTCCAA	eRNA#13A-R	AAGCGCCTTGTGGTTAAAGA
		eRNA#13B-F	ACCCTCTTTCCTCGTCACCT	eRNA#13B-R	GCAAGGCCAATATGACTGAA
ZCCHC2	*MetaEnhancer_804_+*	eRNA#14A-F	CCTATGTGTTCGTGGCTTGA	eRNA#14A-R	TTTGGTGTCCTTTTAACATCCA
		eRNA#14B-F	AAGAACCCAAGCCCAGGAT	eRNA#14B-R	GCTCAGGAAGTTCTGAATCAGTC
		eRNA#14C-F	AAGGACACCAAAAATCTCTGACA	eRNA#14C-R	ATCCTGGGCTTGGGTTCTT
CD38	*MetaEnhancer_1694_+*	eRNA#17A-F	GTAGCATTCTGTGCTCATTTATAC	eRNA#17A-R	TAAATGAGCACAGAATGC
		eRNA#17B-F	CCACTTTTAGGAGTGTATGTACTTGG	eRNA#17B-R	CAAATGTTCTTGTGGCAGCA
APOBEC3D-2	*MetaEnhancer_94_-*	eRNA#18A-F	ATCTGGGTGTGTCTGGGAAA	eRNA#18A-R	GCACACACAGACAGTGACAGG
		eRNA#18B-F	ATGAGCAGCTGTCGAGGAG	eRNA#18B-R	ACACAGACAGTGCCTCAGGA
CCR1	*MetaEnhancer_258_-*	eRNA#23A-F	TCCATTCTTTGTGTTTGGACTG	eRNA#23A-R	TGCGATTATAGAGTGTGAGAAACA
		eRNA#23B-F	CGCAAAGTAGGGAGGTATCTCTT	eRNA#23B-R	TGCAGAAATATTTGAATGACACTTG
TLR7-2	*MetaEnhancer_341_-*	eRNA#24A-F	TATGGAACTGGAGGCACCAT	eRNA#24A-R	TGCAGGTGTTTTATTTTTAAGCA
		eRNA#24B-F	TGACCATCTAGCTCACCATCTG	eRNA#24B-R	TCCCAAACAAGTGGTCTTCA
DMXL1-2	*MetaEnhancer_454_-*	eRNA#26AB-F	TCTCTCTTTCATGGGTCAGTTTC	eRNA#26AB-R	GCAGAAGTTAGCCACCAGGA
		eRNA#26C-F	CAGCCCTGACTTCTCAGCTC	eRNA#26C-R	CAACTGTGTGTGTGTGTGTGTG
WDFY1	*MetaEnhancer_512_-*	eRNA#28A-F	GGCCAAAATTAAAATATGTACAACG	eRNA#28A-R	ACTGTTAAATTAAGTTTGGACTAAAGC
		eRNA#28B-F	CAACGGAGGCTTTAGTCCAA	eRNA#28B-R	GACTGATACAATTATGGCAATACTGA
		eRNA#28C-F	GGTTACATATCAAGTTAGGTTCACTG	eRNA#28C-R	TTTTGGCCTAAAGGTTTTTCA
MYCBP2	*MetaEnhancer_544_-*	eRNA#29A-F	CTGTGTGGAGCTGTGTGATG	eRNA#29A-R	CTCCCAAAAGGCCTCATGTA
		eRNA#29B-F	CTGCTTCGAGTGTGCTCTGT	eRNA#29B-R	TCACACAGCTCCACACAGACT
		eRNA#29C-F	GGCCTTTTGGGAGATACTGA	eRNA#29C-R	GGTTGGAGCCTCAACTCCTA
TNFSF10-2	*MetaEnhancer_734_-*	eRNA#30-F	CCATCTTGGGCCTATCACAC	eRNA#30-R	CTCCACAGAGATGCAGGTCA
IFNb1	*MetaEnhancer_1571_+*	eRNA#34A-F	TTTAGCTCCTCGAAAGGTAGAGA	eRNA#34A-R	TTTAGCTCCTCGAAAGGTAGAGA
		eRNA#34B-F	GCCTGGAAGATGCCTCTGTT	eRNA#34B-R	CTACCTGGCACACACACCAG
IFI16	*MetaEnhancer_1136_+*	eRNA#35-F	CTGCTATCTAGATCTTTG	eRNA#35-R	CTCGCTTGGGTACAACAAT
PARP14	*MetaEnhancer_2972_-*	eRNA#36AB-F	CGACCGTTCCATTCTTGAGT	eRNA#36AB-R	AGAGAAGTTGCAGCGTCAGC
		eRNA#36C-F	CGACCGTTCCATTCTTGAGT	eRNA#36C-R	AGAGAAGTTGCAGCGTCAGC
CD38	*MetaEnhancer_3795_-*	eRNA#37A-F	TGGGCGCTTCATAGGACT	eRNA#37A-R	TCTGGTCTTTTAAAGGGTCTTGA
		eRNA#37BC-F	ACATCTCACCCCCAGACCT	eRNA#37BC-R	CTATGAAGCGCCCAGCTAGA
TNFSF10-3	*MetaEnhancer_2005_+*	eRNA#38AB-F	TGGTGGCAAGATCTCAATCA	eRNA#38AB-R	TCAGGGATGCAGACGAGATA
		eRNA#38C-F	CGGTACAATGCTCTGATTATCTCG	eRNA#38C-R	GGACCATAAGTAATCAGGGATGC
SOCS1	*MetaEnhancer_810_-*	eRNA#39AC-F	TCACGTAAACAAATAAAAGCTTCA	eRNA#39AC-R	CAAAGAAATTGACCCTTCTTGTC
		eRNA#39B-F	TTTTTGAGAGGCCTGAAGGT	eRNA#39B-R	TGAAGCTTTTATTTGTTTACGTGAG
IFI35	*MetaEnhancer_820_-*	eRNA#40-F	ATGGGGTTGAAGCAGGATTT	eRNA#40-R	CAGCCAGTTACAGGGAACTCA
SAMD9	*MetaEnhancer_3039_+*	eRNA#41-F	AAGGCTGATGTGAATCTCTGC	eRNA#41-R	TGGAGCAATCACATGACACA
KIAA0040	*MetaEnhancer_1115_+*	eRNA#42A-F	TCTGCTGCTTCTGAGGGATT	eRNA#42A-R	GATTTAGGAGTTTAATTGAGCAAAAA
		eRNA#42BC-F	CCACGTTGTGGGGAGGAG	eRNA#42BC-R	GAATCCCTCAGAAGCAGCAG
PARP9	*etaEnhancer_2972_-*	eRNA#43A-F	GGCCGTCGTTCTAGTTATGG	eRNA#43A-R	CCGGTTGACACAGCTGGTAG
		eRNA#43B-F	CGACCGTTCCATTCTTGAGT	eRNA#43B-R	TCAGCTGGATGAGCCATAACT
ZBP1	*MetaEnhancer_1228_+*	eRNA#46A-F	ACCAAAAGGTTTAAAAATATCTCG	eRNA#46A-R	ACCTCAGAAGCTCCTGGTCA
		eRNA#46BC-F	CACTCTCATGCAGGGCTGA	eRNA#46BC-R	GTGCTTGGGGTTGTCAGATT
MNDA & ACKR1	*MetaEnhancer_1136_+*	eRNA#47-48A-F	CAGGAGTGGTCTGCCTCTTC	eRNA#47-48A-R	ACTCGCTTGGGTACAACAAT
		eRNA#47-48B-F	TGCTATCTAGATCTTTGACTACCAACC	eRNA#47-48B-R	GAAGAGGCAGACCACTCCTG

Table S9. mRNA primers. Gene name column lists target gene analyzed. Forward primer column contains sequence of the forward primer and Reverse primer column contains sequence of the reverse primer used for detecting mRNA expression.

mRNA primers

Gene name	Forward primer	Reverse primer
HLA-DMA	GATCTAAGGCCACCCTCTCG	GCAGCTCCTTGGTTCTGTTC
HLA-DRA	TCCCGAGCTCTACTGACTCC	TGATAGCCCATGATTCCTGA
HLA-DRB5	CCAGCATGGTGTGTCTGAAG	AGCCAGTGGGGAGCTCAG
TNFSF10	AAGGAAGGGCTTCAGTGACC	GACTGCAGGAGCACTGTGAA
ZFAND6	CCAATCCAGTGGCTCTCCT	CCTCCGTCCGCTGTTTATTA
IRF8	TCGACACCAGCCAGTTCTTC	CTCTTCCCAGCCTCTTCTGC
MLLT6	AGCTCATGGGAGTATGAAGGA	AGTAGACCAGCGGGTTCTCG
PELI1	CCCTCCTTGCGAGTGTATGT	CCGCTGCTGCTAGTGGAG
TLR7	TCCCATCAGAGGCTCATGGA	ATGATTGTCTGTGGCCAGGG
APOBEC3D	AACTTGGCTCACTGCAACCT	GAGTTCGAGACCAGCCTGAC
APOBEC3B	TGCTGGGACACCTTTGTGTA	CCATCCTTCAGTTTCCCTGA
DMXL1	GTGTCCGTTGCAGGACTAGG	CCACGGAGAAGCAGTGGT
ZCCHC2	CCTGAGGGAACACTTGGAGA	AACTTGCACGGCTCTACCTC
CD2AP	GCGCTGAAGAGACTGGTAGG	GCTCCTCCTCCTCCTCCTC
CD38	GGGAGGTGCAGTTTCAGAAC	CGAGGATCAGGACCAGGATA
CCR1	CATCTCCAACCAAGGACCCC	CACACAGTGGGCACATTTTGT
SEMA4D	AACACTCACCGTGAAGGTCTG	CGTCTGGAGTCTGTCCCTTC
WDFY1	GGCCGAAATCCACTCCAG	CTCCTTGGGGATGAGCAG
MYCBP2	GAAGGAGGTCGCTGTCTTTG	ACACACAGCCCTTTTCCAAC
SOCS1	CTGGAGCACTACGTGGCG	AGGGGAAGGAGCTCAGGTAG
IFNB1	TCTCCTAGCCTGTGCCTCTG	GCCATCAGTCACTTAAACAGCA
IFI16	GAATAGGAGCAAGCCAGCAC	AAGTTCCCAGAAACGGAACC
PARP14	TACTTCCAGAGCCCGAAGAG	CGGGTAGAAGAACACCAGGA
CD38	GGGAGGTGCAGTTTCAGAAC	CGAGGATCAGGACCAGGATA
IFI35	AGGGATTTCACGGAAATGAA	AAGGGCTGTGGTCTCTCTCA
SAMD9	CCGTTAAAACCAGAATGAGGA	TGTGGGGAAAACCATCTCTT
KIAA0040	TTCAGGAAGTTGTGCCTGTG	TGTGTTCCCTGCCTTCCTAC
PARP9	CTGCCCTTTCACTGAACTCC	CGAAGGAAGCTGGAGAGCTA
ZBP1	GCATCTATTTCCGGGCTGTA	CTGCAAGGAGTCGGAGAGAC
MNDA	TGGCTCTAACAAGTGCCATT	CACTGTCCGTAAAGCTTTGGA
ACKR1	CTGTCCCATTGTCCCCTAGA	CTGTCGAGGCTGCATAATGA
